# Long-term multidimensional health status of individuals with and without post COVID-19 condition: A cross-sectional study

**DOI:** 10.1371/journal.pone.0352332

**Published:** 2026-07-07

**Authors:** Debbie Gach, Frits H. M. van Osch, Joop P. van den Bergh, Rein Posthuma, Hester A. Gietema, Martijn D. de Kruif, Annemie M. W. J. Schols, Rosanne J. H. C. G. Beijers, on behalf of the COVID CLIMATE Consortium

**Affiliations:** 1 Department of Respiratory Medicine, NUTRIM, Institute of Nutrition and Translational Research in Metabolism, Maastricht University Medical Centre+, Maastricht, the Netherlands; 2 Department of Clinical Epidemiology, VieCuri Medical Centre, Venlo, The Netherlands; 3 Department of Epidemiology, GROW, Research Institute for Oncology and Reproduction, Maastricht University Medical Centre+, Maastricht, the Netherlands; 4 Department of Internal Medicine, VieCuri Medical Centre, Venlo, The Netherlands; 5 Department of Internal Medicine, NUTRIM, Institute of Nutrition and Translational Research in Metabolism, Maastricht University Medical Centre+, Maastricht, the Netherlands; 6 Ciro+, Department of Research & Development, Ciro+ Horn, Horn, The Netherlands; 7 Department of Respiratory Medicine, Maastricht University Medical Centre, Maastricht, The Netherlands; 8 Department of Radiology and Nuclear Medicine, GROW, Research Institute for Oncology and Reproduction, Maastricht University Medical Centre+, Maastricht, the Netherlands; 9 Department of Respiratory Medicine, Zuyderland Medical Centre, Heerlen, The Netherlands; Museo Storico della Fisica e Centro Studi e Ricerche Enrico Fermi, ITALY

## Abstract

Post COVID-19 condition (PCC) significantly affects health-related quality of life (HRQoL), but involvement and interconnectivity of different health dimensions is still underexplored. This study aims to characterize the multidimensional health status of individuals with and without PCC after at least one year follow-up. Hospitalized and non-hospitalized COVID-19 patients were included from three hospitals in the Netherlands. HRQoL, pulmonary and metabolic health, muscle strength, physical capability, symptoms, psychological- and social wellbeing, and cognitive function were measured using validated objective and subjective methodology. Presence of PCC was based on self-report. 139 participants were included, of which 87 with PCC and 52 without PCC. HRQoL was lower in the PCC group compared to the non-PCC group (EQ-5D: *p* = 0.005; VAS: *p* < 0.001). Individuals with PCC also more frequently reported fatigue, anxiety/depression, stress and loneliness, alongside lower subjective cognitive functioning and sleep quality (*p* < 0.05). Inspiratory muscle strength and exercise capacity (*p* = 0.024 and *p* = 0.007) were lower in the PCC group than the non-PCC group. Pulmonary function and residual CT-abnormalities, body composition, cardiometabolic risk, expiratory- and peripheral muscle strength, mobility, physical activity, and cognitive function tests were not different between groups. Perceived health burden in PCC is reflected by lower health status, more frequently reported symptoms of fatigue and poor sleep quality, and lower psychological- and social wellbeing and subjective cognitive functioning compared to non-PCC. These differences are only partially reflected in objectively assessed dimensions of muscle strength and physical capability, but not in pulmonary/metabolic health and cognitive function. The findings indicate a discordance between subjective health burden and currently available objective assessments in individuals with PCC.

## Introduction

Since its emergence in late 2019, the COVID-19 pandemic, caused by the severe acute respiratory syndrome coronavirus 2 (SARS-CoV-2), has resulted in 778 million reported cases worldwide [1]. Although many individuals recovered from the acute phase of COVID-19, a substantial proportion of patients continue to experience symptoms, termed as post COVID-19 condition (PCC). The World Health Organization defines PCC as symptoms that occur at least three months after probable or confirmed SARS-CoV-2 infection, that persist for at least two months and cannot be explained by an alternative diagnosis [2,3]. Currently, the diagnosis of PCC relies solely on clinical assessment through detailed symptom characterization, in the absence of objective diagnostic tests [4]. The global pooled prevalence of PCC has been estimated to be 54% and 34% in hospitalized and non-hospitalized COVID-19 patients, respectively, with the most common symptoms being dyspnoea on exertion, concentration difficulties, and fatigue [5,6]. The exact prevalence and health burden of PCC however remains unclear due to heterogeneity in study populations (i.e., vaccinated vs unvaccinated), follow-up timing, and PCC definitions.

Beyond the most common symptoms, PCC has been demonstrated to encompass a wide range of psychological manifestations such as anxiety, depression, post-traumatic stress disorder, and sleep disturbances [7,8]. Next to these subjective health effects, a variety of objective health impairments has been identified in both post-hospitalized and non-hospitalized patients with PCC, including reduced exercise capacity, decreased respiratory and overall muscle strength, as well as diminished physical activity levels, up to one year after infection [9–12]. Additionally, persisting pulmonary sequelae such as diffusion capacity impairments and pulmonary fibrosis have commonly been observed, particularly among previously hospitalized COVID-19 patients [13].

The broad spectrum of health complaints reported by PCC patients impairs their daily activities and overall quality of life [14,15]. The diverse symptomatology also underscores the multifaceted nature of PCC, highlighting the need for a comprehensive health assessment across multiple health domains to investigate interconnectivity and optimize long-term outcomes after tailored intervention [16]. To date, few studies have investigated PCC from a multidimensional perspective [17,18]. These studies have largely focused on post-hospitalized COVID-19 patients and consistently demonstrate continued impairments in various health domains including pulmonary function abnormalities (i.e., reduced diffusion capacity), mental health issues (i.e., depression and anxiety), reduced exercise capacity, and ongoing symptoms such as fatigue and dyspnoea, up to two years post-infection [17–20]. Although recent studies have adopted comprehensive multidimensional health assessments, these have not consistently compared individuals with and without PCC. Given the heterogeneity and complexity of PCC, further studies employing comprehensive multidimensional health approaches integrating both subjective and objective measures are still essential to advance and deepen our understanding of its long-term health consequences.

This study therefore aimed to characterize the multidimensional health status of individuals (previously hospitalized and non-hospitalized) with self-reported PCC, compared to those without self-reported PCC, at least one year after a SARS-CoV-2 infection, using validated objective and subjective methodology.

## Methods

### Study design and population

A cross-sectional study was designed including individuals aged >18 years, with proven SARS-CoV-2 infection (positive PCR test, proven serology, or a CORADS score ≥4) [21], who were hospitalized or referred due to persisting symptoms to the post COVID-19 outpatient clinic at one of the following hospitals in, the Netherlands; Maastricht University Medical Centre+ (MUMC+), VieCuri Medical Centre, and Zuyderland Medical Centre. Subjects were invited for a multidimensional health assessment at the MUMC+ clinical research unit at least one year after the SARS-CoV-2 infection between 17/02/2022 and 11/03/2024. The minimum follow-up duration of one-year was made to ensure that long-term health consequences were adequately captured as informed by previous evidence from infections with related coronaviruses, such as Severe Acute Respiratory Syndrome, where persistent impairments beyond one year have been reported.

The study was approved by the medical ethical research committee of the Maastricht University Medical Centre+ (NL76949.068.21/MEC21–040) and conducted in accordance with the Declaration of Helsinki [22]. All participants gave written informed consent before enrolling in the study.

### Data collection

#### Study visit.

The multidimensional health assessment was generally performed in one day, and occasionally in two days depending on the preference of the participant. Participants were instructed to arrive in a fasted state (abstaining from food, beverages, and smoking after 8 pm on the evening before the visit). Baseline and hospitalization characteristics as well as date of primary SARS-CoV-2 infection were collected via questionnaires and confirmed with electronic medical records of the participants. The respective COVID-19 wave was based on the date of the primary infection, and categorized as followed (first, March-June 2020; second, July-December 2020; third, January-September 2021; fourth, October 2021-March 2022). Additionally, the Charlson Comorbidity Index (CCI) was calculated for each patient [23]. Table 1 provides an overview of the measurements with its corresponding reference values included in the multidimensional health assessment.

**Table 1:** Overview of the multidimensional health measurements.

Outcome	Measurement	Reference value
**Quality of life**		
Health-related quality of life	EQ-5D-3L/ EQ-5D VAS*	Sex-, age-, and population specific norms [24,25]
**Pulmonary health**		
Pulmonary function	Pre-bronchodilator spirometry and body plethysmography	Sex-, age-, height-, and population specific reference [26]
Diffusion capacity	The single-breath method	Sex-, age-, height-, and population specific reference [27]
COVID-19 related residual CT-abnormalities	Chest CT scan	–
**Metabolic health**		
Body composition	DXA scan	–
Glucose levels	Blood sampling	–
LDL cholesterol levels	Blood sampling	–
HDL cholesterol levels	Blood sampling	–
Total triglyceride levels	Blood sampling	–
Waist circumference	Waist circumference	–
Blood pressure	Omron blood pressure monitor	–
**Muscle strength**		
Mouth pressure	MicroRPM monitor	Sex- and age specific reference [28]
Handgrip strength	Hydraulic dynamometer	–
Quadriceps muscle strength	Biodex	–
**Physical capability**		
Mobility	SPPB	High performance: 10–12 [29] Moderate performance: 7–9 Low performance: 0–6
Cardiorespiratory fitness	6MWT	Sex-, age-, height-, and weight specific reference [30]
Physical activity level	Accelerometry	–
**Symptoms**		
Rate of dyspnoea	mMRC scale*	Higher score (0–4)> more dyspnoea [31]
Fatigue levels	CIS*	Severe fatigue: ≥ 35 [32]
Sleep quality	PSQI*	Sleep problems: > 5 [33]
**Psychological- and social wellbeing**		
Anxiety and depression	HADS*	Anxiety and depression: ≥ 10 [34]
Stress	PSS*	Higher score (0–40)> greater stress [35]
Social support	MSPSS*	Higher score (0–7)> greater support [36]
Loneliness	LS*	Higher score (0–11)> more loneliness [37]
**Cognitive function**		
Objective cognitive function	MoCA	Cognitive impairment: < 26 [38]
Subjective cognitive function	CFQ*	Cognitive failure: ≥ 46 [39]

*Questionnaire. Abbreviations: CFQ, Cognitive Failure Questionnaire; CIS, Checklist Individual Strength; CT, computed tomography; DXA, dual-energy x-ray absorptiometry; EQ-5D-3L, three-level EuroQol five-dimensions; HADS, Hospital Anxiety and Depression Scale; HDL, high-density lipoprotein; LDL, low-density lipoprotein; LS, Loneliness Scale; mMRC, modified Medical Research Council; 6MWT, 6-minute walking test; MoCA, Montreal Cognitive Assessment; MSPSS, Multidimensional Scale of Perceived Social Support; PSQI, Pittsburgh Sleep Quality Index; PSS, Perceived Stress Scale; SPPB, Short Physical Performance Battery; VAS, Visual Analogue Scale.

### Multidimensional health assessment

#### Quality of life.

Health-related quality of life (HRQoL) of participants was measured by the three-level EuroQol five-dimensions questionnaire (EQ-5D-3L) and the EQ-5D visual analogue scale (VAS) [24,25]. For the EQ-5D-3L, participants had to describe their health among five domains: mobility, self-care, usual activities, pain/discomfort and anxiety/depression, using a three-level scale (1: no problems, 2: some problems, and 3: severe problems). The sum of level 2 and 3 were calculated to evaluate the percentage of participants reporting problems in each specific domain. For the EQ-5D VAS, participants had to rate their health on that day on a scale of 0–100 (100 is best health state).

#### Pulmonary health.

Pulmonary function tests were performed according to European Respiratory Society guidelines [26,40]. Pre-bronchodilator spirometry was performed to assess forced expiratory volume in one second (FEV_1_) and forced vital capacity (FVC). The single-breath method was used to determine diffusion capacity of the lungs for carbon monoxide (DLCO) and body plethysmography was performed to measure total lung capacity (TLC) [27]. An unenhanced chest computed tomography (CT) scan (SOMATOM Force, Siemens Healthineers, Erlangen, Germany) was performed to assess COVID-19 related residual abnormalities. Interpretation of images was done by one experienced radiologist.

#### Metabolic health.

Body composition was assessed using a whole-body dual-energy x-ray absorptiometry (DXA) scan (Discovery A, Hologic inc., Marlborough, MA, USA). Fat free mass index (FFMI) was calculated by fat free mass (FFM)/height^2^ and skeletal muscle mass index (SMI) by appendicular lean mass/height^2^. Blood samples were collected and cardiometabolic risk markers were measured including glucose, triglycerides, high- and low-density lipoprotein cholesterol using the Cobas Pro Roche analyser (Roche Diagnostics, Mannheim, Germany). Additionally, blood pressure (Omron Healthcare, Hamburg, Germany) and waist circumference (Seca 201, Seca GmbH & Co. KG, Hamburg, Germany) were measured.

#### Muscle strength.

Respiratory muscle strength was measured by maximal inspiratory- and expiratory pressure (MIP and MEP) according to current guidelines using the MicroRPM monitor^TM^ (MD Spiro, Lewiston, United States) [28,41]. Upper extremity strength was assessed by handgrip strength (HGS) in the dominant hand using the Jamar Hydraulic Hand Dynamometer (Performance Health, Warrenville, Illinois, United States). Maximum quadriceps isometric muscle strength of the dominant leg was measured using the Biodex system 4 (Biodex Corporation, Shirley, New York, United States). Patients’ knee of the dominant leg was placed at a 60 degrees angle and patients were instructed to maximally contract three times for five seconds each.

#### Physical capability.

The Short Physical Performance Battery (SPPB) test, consisting of three types of physical manoeuvres; the balance test, the gait speed test, and the chair stand test, was used to assess mobility [29,42]. Exercise capacity was measured by the six-minute walking test (6MWT), which was performed according to American Thoracic Society guidelines [30,43]. At the end of the study visit, an accelerometer (activPAL^TM^, Pal Technologies Ltd, Glasgow, Scotland) was placed on the midline of the right thigh to measure daily physical activity levels for seven days.

#### Symptoms.

The modified Medical Research Council (mMRC) scale was used to determine the severity of dyspnoea experienced in daily life [31]. Fatigue levels were measured using the Checklist Individual Strength (CIS) [32,44]. The Pittsburgh Sleep Quality Index (PSQI) was used to assess sleep quality [33].

#### Psychological and social wellbeing.

The Hospital Anxiety and Depression scale (HADS) was used to determine levels of anxiety and depression [34,45]. The Perceived Stress Scale (PSS) was used to assess the degree to which situations in one’s life are perceived as stressful [35]. Perceived social support across three domains: family, friends, and significant others, was measured by the Multidimensional Scale of Perceived Social Support (MSPSS) [36]. Lastly, the Loneliness Scale (LS) was used to determine perceived levels of loneliness [37].

#### Cognitive function.

The Montreal Cognitive Assessment (MoCA) tool was used to assess objective cognitive functioning [38]. Subjective cognitive function was assessed using the Cognitive Failure Questionnaire (CFQ) [39].

#### Follow-up call.

Due to the evolving understanding of PCC, a follow-up call was conducted with each participant after the study visit at a mean interval of 13 ± 5.7 months, to complete additional post COVID-19 questions, which were constructed by an expert’s team of COVID-19 specialists, including an internist, pulmonologist, and post COVID-19 researchers. The questions assessed symptom status and perceived PCC, and included: (1) ‘Did you experience any symptoms at the time of the study visit?’ *(if yes)*, (2) ‘Do you believe these symptoms were related to the COVID-19 infection?’ *(if yes)*, (3) ‘Do you think you had PCC at the time of the study visit?’. Participants were classified as having self-reported PCC if they responded ‘yes’ to all three preceding questions. The PCC classification, based on self-reported symptoms, reflects real-world clinical reporting but may differ from formal diagnostic frameworks such as the WHO-based criteria. These criteria were not yet available at the time the study was designed and could therefore not be applied in the present study.

### Statistical analysis

Data normality was checked with visual histograms as well as Q-Q plots and Shapiro–Wilk tests. For comparison of patient characteristics and multidimensional health dimensions between individuals with and without PCC, a Chi-Square test was calculated for categorical/ordinal variables and an independent samples T-test or Mann–Whitney U-test was performed for continuous variables depending on normality. Post hoc pairwise comparisons between categories of ordinal variables were performed using Bonferroni-adjusted *p*-values to control for multiple testing. Pearson correlations were calculated to investigate the associations between HRQoL and multidimensional health dimensions in the total cohort. Analyses were performed using IBM SPSS statistics, version 28. A *p-*value <0.05 was considered statistically significant.

## Results

### Baseline characteristics

A total of 139 evaluable individuals participated in the study, of whom 87 self-reported PCC. Mean age of individuals with PCC was 59 ± 14 years with the majority being male (68%; Table 2). A lower number of individuals with PCC was observed during the second wave compared to those without (*p* = 0.011). The PCC group had fewer former smokers (*p* = 0.008), lower CCI scores (*p* = 0.009), and fewer classifications of a severe CCI score (*p* = 0.012), than the non-PCC group. Higher hospitalization rates were observed in the non-PCC group (*p* = 0.010), while ICU admission rates were comparable between both groups.

**Table 2:** Baseline characteristics of the individuals with and without self-reported PCC.

Baseline characteristics	Individuals with self-reported PCC (n = 87)	Individuals without self-reported PCC (n = 52)	*p*-value
Waves			**0.020**
*First (Feb-June ‘20)*	30 (35)	18 (34)	
*Second (July-Dec ’20)*	10 (12)	15 (29)*	
*Third (Jan-Sept ’21)*	20 (23)	4 (8)	
*Fourth (Oct ’21-March ’22)*	26 (30)	15 (29)	
Age in years	59 ± 14	63 ± 12	0.063
Male	59 (68)	37 (71)	0.680
Smoking status			**0.016**
*Never*	43 (49)	16 (31)	
*Former*	40 (46)	36 (69)**	
*Current*	4 (5)	0 (0)	
CCI score^a^	2 (1–3)	3 (2–5)	**0.009**
*Severe (CCI score ≥5)*	8 (9)	13 (25)	**0.012**
**Hospital stay**			
Hospital admission	52 (60)	42 (81)	**0.010**
Length of hospital stay in days^ab^	9 (4–22)	10 (4–17)	0.862
ICU admission	18 (21)	16 (31)	0.780
Length of ICU stay in days^ac^	16 (8–35)	9 (4–19)	0.196
**Treatments during hospital stay**			
Nasal oxygen therapy	46 (88)	37 (88)	0.727
Non-invasive ventilation	16 (31)	9 (21)	0.513
Invasive ventilation	12 (23)	7 (17)	0.225
**Follow-up**			
Time since initial infection in days^a^	765 (519–1121)	826 (563–1193)	0.558
Vaccination status	81 (93)	48 (92)	≥0.999
Any form of rehabilitation	80 (92)	45 (87)	0.305

Data are shown as mean±SD or n (%) unless indicated otherwise. ^a^Median (IQR). ^b^Assessed in 49/40 of the individuals with and without self-reported PCC due to hospital transfers. ^c^Assessed in 16/16 of the individuals with and without self-reported PCC due to hospital transfers. Bold indicates a significant difference between individuals with and without self-reported PCC, *p* < 0.05. *Indicates a significant difference between individuals with and without self-reported PCC, *p* = 0.011. **Indicates a significant difference between individuals with and without self-reported PCC, *p* = 0.008. Abbreviations: CCI, Charlson Comorbidity Index; ICU, intensive care unit; PCC, post COVID-19 condition.

### Quality of life

**Fig 1:**
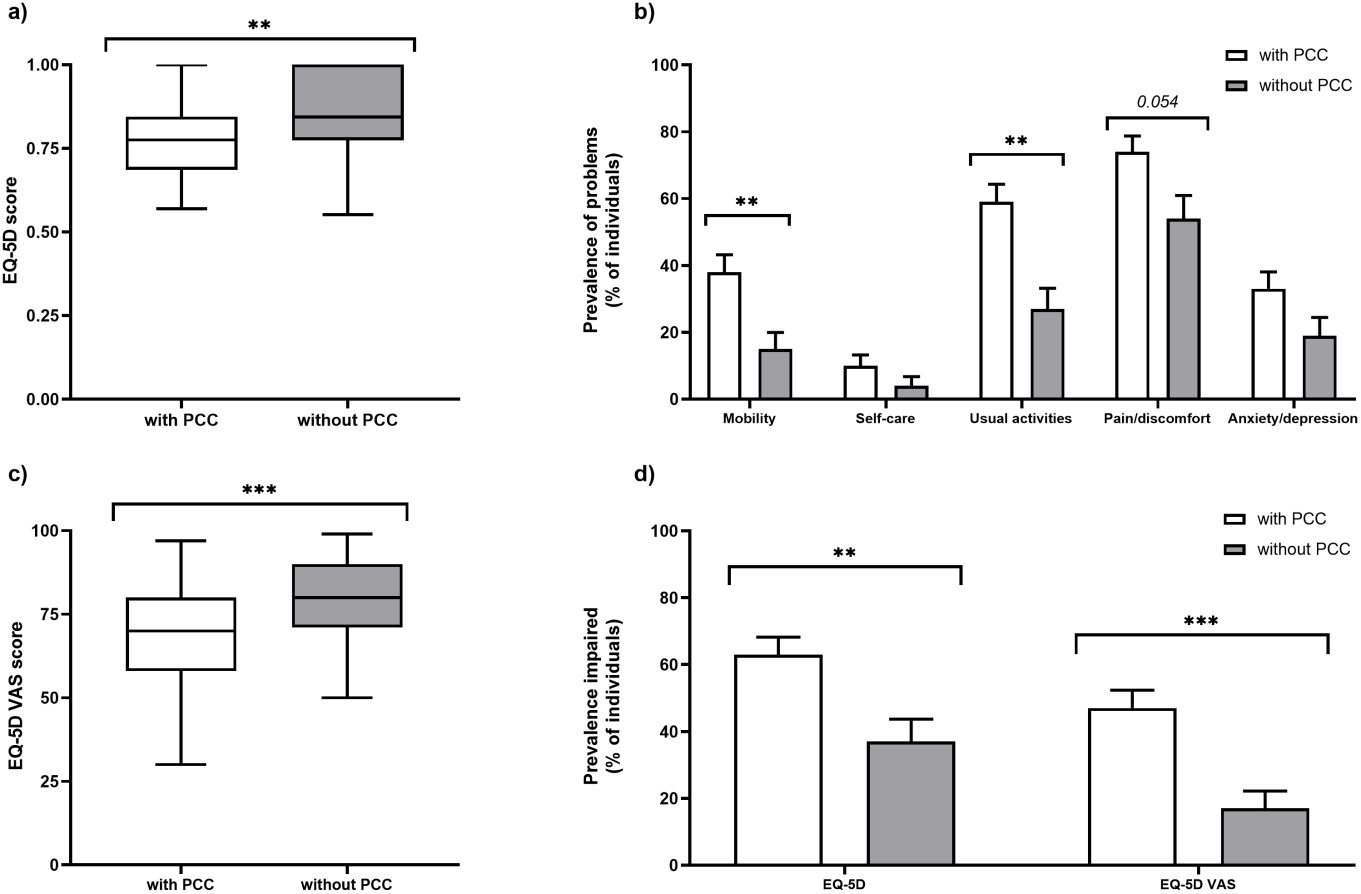
Health-related quality of life in the individuals with and without self-reported PCC (n = 87/52). **Note:** A: EQ-5D scores stratified by self-reported PCC. B: EQ-5D categories stratified by self-reported PCC. C: EQ-5D VAS scores stratified by self-reported PCC. D: EQ-5D (VAS) impairments stratified by self-reported PCC. Data are shown as median (IQR) for continuous variables and n (%) ±SD for categorical variables. *Significantly different between individuals with and without self-reported PCC, *p* < 0.05, **p < 0.01, ***p < 0.001. Abbreviations: EQ-5D, EuroQol five-dimensions; PCC, post COVID-19 condition; VAS, Visual Analogue Scale.

A lower HRQoL was reported by individuals with PCC (EQ-5D: 0.78 (0.69–0.84) vs 0.84 (0.78–1.00); *p* = 0.005 and VAS: 70 (60–84) vs 80 (71–90); *p* < 0.001, respectively; Fig 1ac). The PCC group showed higher prevalence of problems in domains of mobility (38% vs 15%; *p* = 0.005) and usual activities (59% vs 27%; *p* = 0.001) compared to the non-PCC group. Additionally, both groups reported the highest prevalence of problems in the domain of pain/discomfort (Fig 1b). Impairments in HRQoL were also more often reported among individuals with PCC than those without (EQ-5D: 63% vs 37%; *p* = 0.002 and VAS: 47% vs 17%; *p* < 0.001, respectively; Fig 1d).

### Pulmonary/metabolic health, muscle strength and physical capability

**Fig 2:**
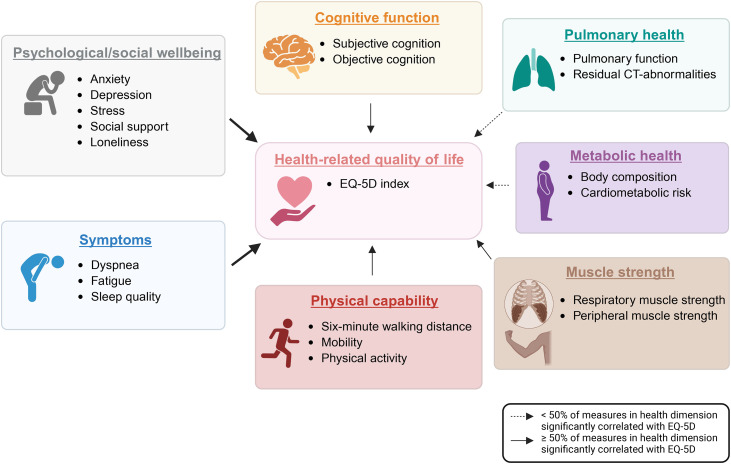
Correlations between health-related quality of life and multidimensional health dimensions (n = 139). **Note:** Thick arrow represents a moderate correlation (*r* = 0.400-0.599) between health dimension and health-related quality of life. Thin arrow represents a weak correlation (*r* = 0.000-0.399) between health dimension and health-related quality of life. Abbreviations: CT, computed tomography; EQ-5D, EuroQol five-dimensions. Reprinted from [Biorender] under a CC BY license, with permission from [Debbie Gach], original copyright [2026].

No significant correlation was found between HRQoL and measures of pulmonary and metabolic health (Fig 2/Supporting information S1 Table). However, all measures of muscle strength and physical capability showed a weak positive correlation with HRQoL (*r* = 0.206–0.326 and *r* = 0.169–0.402, respectively, *p* < 0.05). Mean MIP and mean 6MWD in %predicted was lower in the PCC group than the non-PCC group (*p* = 0.024 and *p* = 0.007; Table 3). Pulmonary function, residual CT-abnormalities, body composition and cardiometabolic risk were comparable between both groups. Likewise, MEP, upper- and lower extremity strength, mobility scores, and physical activity levels did not differ between the individuals with and without PCC.

**Table 3:** Pulmonary/metabolic health, muscle strength and physical capability in the individuals with and without self-reported PCC.

	Individuals with self-reported PCC (n = 87)	Individuals without self-reported PCC (n = 52)	*P*-value
**Pulmonary health**			
Pulmonary function			
FEV_1_ in %pred^b^	106.5 ± 19.5	107.6 ± 24.3	0.783
FVC in % pred^b^	93.9 ± 18.8	96.2 ± 17.7	0.478
FEV_1_/FVC ratio in L^b^	0.86 ± 0.17	0.89 ± 0.13	0.252
DLCO in %pred	88.9 ± 16.2	87.0 ± 17.3	0.528
TLC in %pred	88.9 ± 13.6	90.6 ± 9.8	0.428
Pulmonary features			
Residual CT-abnormalities	33 (38)	23 (44)	0.295
**Metabolic health**			
Body composition ^c^			
BMI in kg/m^2^	30.3 ± 5.4	29.4 ± 4.9	0.326
FFMI in kg/m^2^	20.8 ± 3.0	20.8 ± 2.7	0.973
SMI in kg/m^2^	8.5 ± 1.4	8.4 ± 1.3	0.709
BMD in g/cm^2^	1.1 ± 0.1	1.1 ± 0.1	0.334
Cardiometabolic risk			
Glucose in mmol/L^d^	5.4 (5.0–6.1)	5.7 (5.2–6.5)	0.178
LDL cholesterol in mmol/L^e^	3.1 ± 0.9	2.9 ± 0.9	0.361
HDL cholesterol in mmol/L^c^	1.3 ± 0.4	1.4 ± 0.3	0.944
Triglycerides in mmol/L^c^	1.6 (1.2–2.4)	1.6 (1.2–2.3)	0.934
Waist circumference in cm	105.2 ± 15.7	106.7 ± 15.7	0.601
Systolic blood pressure in mm Hg	132.6 ± 16.0	134.5 ± 16.0	0.490
Diastolic blood pressure in mm Hg	82.0 ± 10.3	81.2 ± 11.0	0.669
**Muscle strength**			
Respiratory muscle strength ^f^			
MIP in %pred	91.1 ± 29.6	102.9 ± 29.0	**0.024**
MEP in %pred	85.1 ± 32.2	94.5 ± 41.7	0.141
Upper extremity strength			
Max. HGS dominant in kg	36.8 ± 11.7	37.8 ± 11.6	0.619
Lower extremity strength			
Peak torque in Nm	126.3 ± 50.3	130.0 ± 45.1	0.666
**Physical capability**			
Mobility			
SPPB score			0.088
*High performance*	73 (84)	50 (96)	
*Moderate performance*	13 (15)	2 (4)	
*Low performance*	1 (1)	0 (0)	
Cardiorespiratory fitness			
6MWD in m	476 ± 89	500 ± 94	0.136
6MWD in %pred	71.7 ± 12.4	77.8 ± 12.2	**0.007**
Physical activity level ^c^			
Total step count per day	7762 ± 3078	8098 ± 4505	0.471
Activity Score in MET.h	33.5 ± 1.3	33.7 ± 1.9	0.577

Data are shown as mean±SD or n (%) unless indicated otherwise. ^a^Median (IQR). ^b^Reported in 78/51 of the individuals with and without self-reported PCC due to invalid measurements. ^c^Assessed in 85/51 of the individuals with and without self-reported PCC because of practical issues. ^d^Assessed in 86/50 of the individuals with and without self-reported PCC because of practical issues. ^e^Assessed in 84/50 of the individuals with and without self-reported PCC because of practical issues. ^f^Assessed in 86/51 of the individuals with and without self-reported PCC because of practical issues. Bold indicates a significant difference between individuals with and without self-reported PCC, *p* < 0.05. Abbreviations: BMD, bone mineral density; BMI, body mass index; CT, computed tomography; DLCO, diffusion capacity of the lungs for carbon monoxide; FEV_1_, forced expiratory volume in one second; FFMI, fat free mass index; FVC, forced vital capacity; HDL, high-density lipoprotein; HGS, handgrip strength; LDL, low-density lipoprotein; MEP, maximal expiratory pressure; MIP, maximal inspiratory pressure; 6MWD, 6-minute walking distance; PCC, post COVID-19 condition; SMI, skeletal muscle mass index; SPPB, Short Physical Performance Battery; TLC, total lung capacity.

### Symptoms

**Fig 3:**
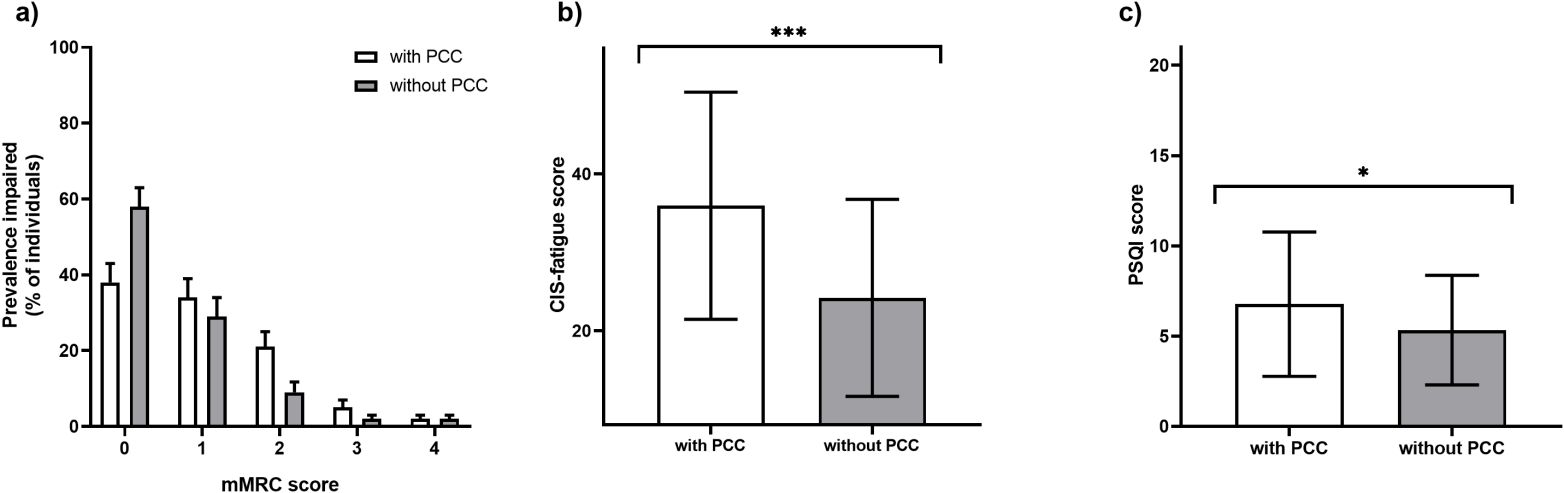
Symptoms in the individuals with and without self-reported PCC (n = 87/52). **Note:** A: mMRC scores stratified by self-reported PCC. B: CIS scores stratified by self-reported PCC. C: PSQI scores stratified by self-reported PCC. Data are shown as mean±SD or median (IQR) for continuous variables depending on normality and n (%) ±SD for categorical variables. *Significantly different between individuals with and without self-reported PCC, *p* < 0.05, **p < 0.01, ***p < 0.001. Abbreviations: CIS, Checklist Individual Strength; mMRC, modified Medical Research Council; PCC, post COVID-19 condition; PSQI, Pittsburgh Sleep Quality Index.

All symptoms showed a moderate negative correlation with HRQoL (*r* = −0.512 – −0.437, *p* < 0.001; Fig 2/Supporting information S1 Table). The PCC group showed higher CIS-fatigue and PSQI scores compared to the non-PCC group (36 ± 14 vs 24 ± 13; *p* < 0.001 and 7 ± 4 vs 5 ± 3; *p* = 0.026, respectively; Fig 3bc). Additionally, a higher proportion of individuals with PCC had impaired CIS-fatigue scores (55% vs 19%; *p* < 0.001; Supporting information S1a Fig). mMRC scores were comparable between groups (Fig 3a).

### Psychological- and social wellbeing

**Fig 4:**
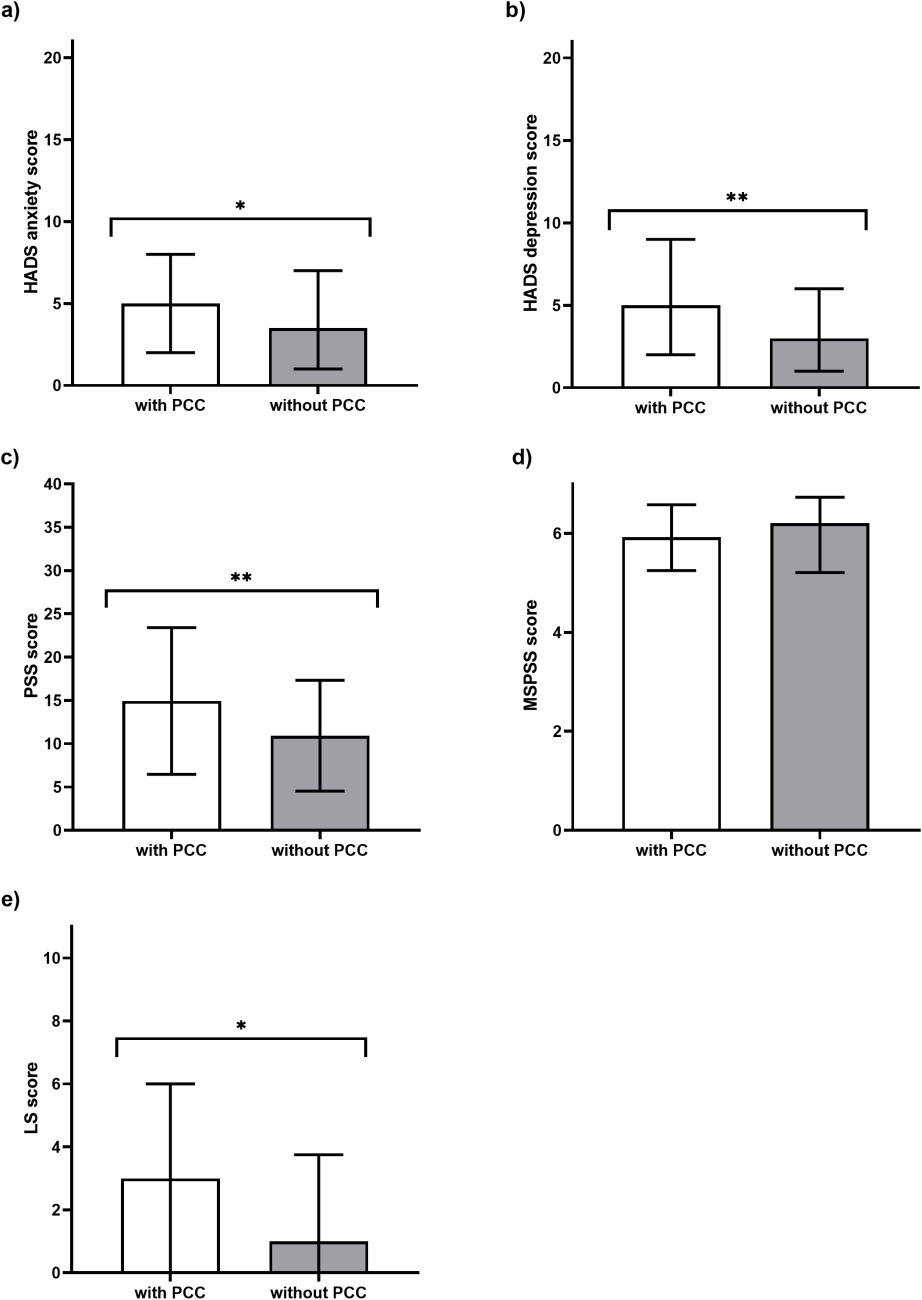
Psychological- and social wellbeing in the individuals with and without self-reported PCC (n = 87/52). **Note:** A: HADS anxiety scores stratified by self-reported PCC. B: HADS depression scores stratified by self-reported PCC. C: PSS scores stratified by self-reported PCC. D: MSPSS scores stratified by self-reported PCC. E: LS scores stratified by self-reported PCC. Data are shown as mean±SD or median (IQR) for continuous variables depending on normality. *Significantly different between individuals with and without self-reported PCC, *p* < 0.05, **p < 0.01, ***p < 0.001. Abbreviations: HADS, Hospital Anxiety and Depression Scale; LS, Loneliness Scale; MSPSS, Multidimensional Scale of Perceived Social Support; PCC, post COVID-19 condition; PSS, Perceived Stress Scale.

Poorer psychological- and social wellbeing measures were moderately associated with lower HRQoL (*r* = −0.551–0.451, *p* < 0.001; Fig 2/Supporting information S1 Table). Higher HADS anxiety and depression scores were observed in the PCC group (*p* < 0.05; Fig 4ab), with a greater proportion reporting abnormal HADS depression scores compared to the non-PCC group (21% vs 4%; *p* = 0.006; Supporting information S2b Fig). Similarly, PSS and LS scores were higher among individuals with than without PCC (*p* < 0.05; Fig 4ce). Mean MSPSS scores were not different between groups (Fig 4d).

### Cognitive function

**Fig 5:**
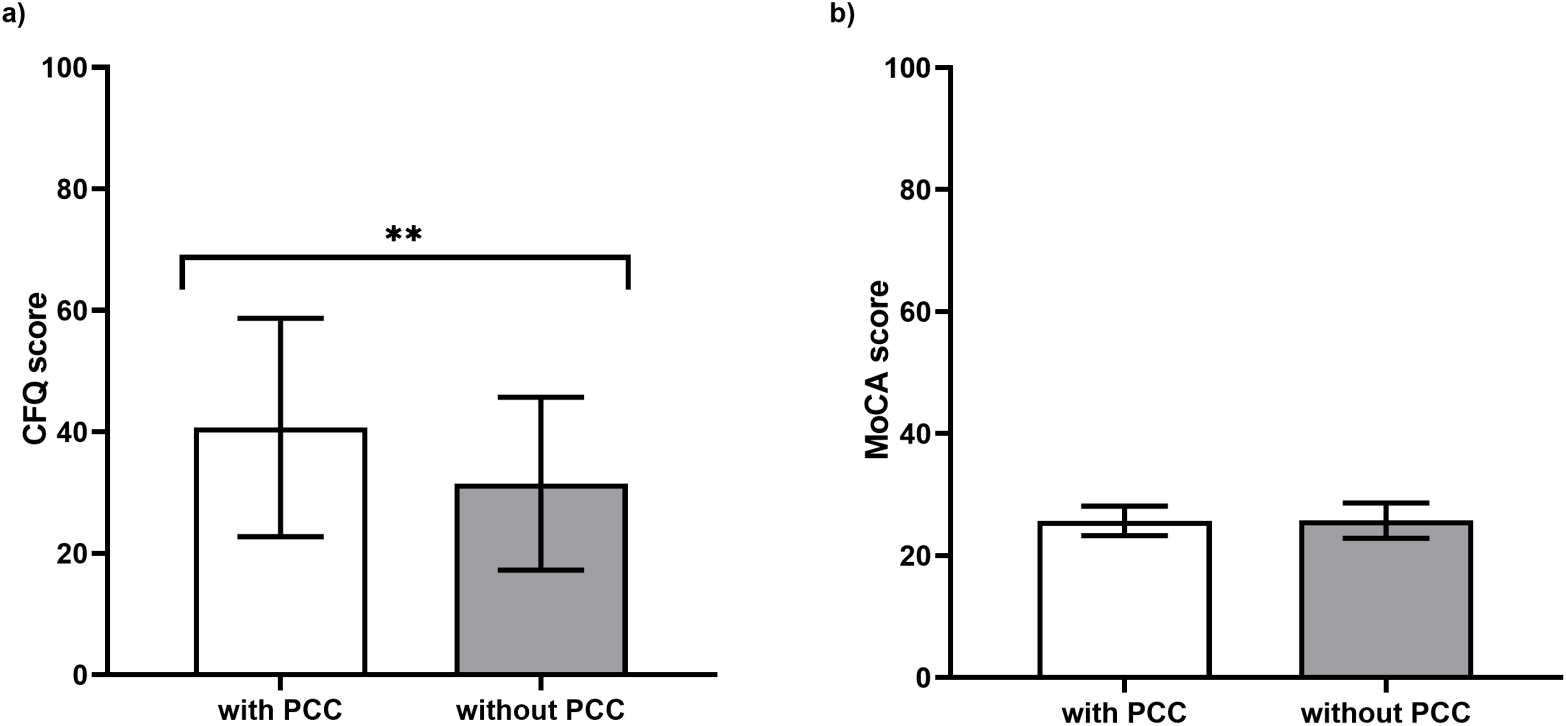
Cognitive function in the individuals with and without self-reported PCC (n = 87/52). **Note:** A: CFQ scores stratified by self-reported PCC. B: MoCA scores stratified by PCC. Data are shown as mean±SD or median (IQR) for continuous variables depending on normality. *Significantly different between individuals with and without self-reported PCC, *p* < 0.05, **p < 0.01, ***p < 0.001. Abbreviations: CFQ, Cognitive Failure Questionnaire; MoCA, Montreal Cognitive Assessment; PCC, post COVID-19 condition.

Worse objective and subjective cognition weakly correlated with lower HRQoL (*r* = 0.241 and *r* = −0.392, *p* < 0.01; Fig 2/Supporting information S1 Table). Mean CFQ score was 41 ± 18 in the PCC group, with 36% reporting an abnormal CFQ score, which were both higher than the non-PCC group (31 ± 14 and 14%; *p* < 0.01, respectively; Fig 5a and Supporting information S3a Fig). Mean MoCA scores and the proportion of individuals with an impaired MoCA score were similar between groups (Fig 5b and Supporting information S3b Fig).

## Discussion

This comprehensive prospective observational cohort study showed that individuals with PCC documented a lower HRQoL as compared to those without PCC. No differences in pulmonary/metabolic health and most measures of muscle strength and physical capability dimensions were detected, except for a lower MIP and 6MWD %predicted in the individuals with PCC. The PCC group reported higher levels of fatigue and lower sleep quality than the non-PCC group. Additionally, poorer subjective cognitive functioning and psychological- and social wellbeing, reflected by greater anxiety, depression, stress, and loneliness, were observed in the PCC group.

Results of the current study confirm previous research on the negative impact of PCC on HRQoL of both hospitalized and non-hospitalized COVID-19 patients up to two years after infection [14,15,19,20]. The lower EQ-5D index and EQ-5D VAS scores in the PCC group are considered meaningful according to current literature in other diseases [46]. Together, these findings validate the notion that reduced HRQoL constitutes a key and persistent feature of PCC.

In our cohort, approximately half of the individuals with PCC reported clinically problematic fatigue, which is in line with existing literature [8,19,20,47–49]. These observations suggest a potential overlap with post-viral chronic fatigue syndromes, as similar patterns were reported during the recovery phase of severe acute respiratory syndrome up to four years after infection [50]. In addition, numerous long-term studies in both post-hospitalized and non-hospitalized COVID-19 patients have highlighted the psychological (e.g., anxiety, depression, and stress) and cognitive effects of PCC at two years follow-up, similar to our results [8,19,20,47–49]. We observed discrepancies between self-reported cognitive impairments and objectively assessed cognitive function. This disparity could be explained by individual differences in symptom perception, as well as insufficient understanding of the underlying aetiology of PCC symptoms, as the exact pathophysiological mechanisms are still unknown. While viral persistence, immune dysregulation, and endothelial inflammation with immune thrombosis have been proposed [2,51], further research is required to validate these mechanisms using specific biomarkers. In addition, differences in measurement sensitivity may contribute to this discrepancy. The MoCA, as a brief screening tool for global cognitive impairment, may be insufficiently sensitive to detect subtle or domain-specific deficits, particularly in attention, executive functioning, and cognitive fatigability, which are commonly reported in PCC. In contrast, self-reported measures may better capture subtle and fluctuating cognitive difficulties in daily life. Moreover, subjective cognitive complaints may be influenced by co-occurring factors such as fatigue, sleep disturbances, and psychological distress, potentially contributing to the perception of cognitive dysfunction in the absence of detectable impairments on brief objective measures. Together, this underscores the importance of a multidimensional approach to cognitive assessment in PCC.

Interestingly, our results demonstrated that individuals with PCC had fewer (severe) comorbidities and were less often hospitalized than those without. This contrasts with other studies suggesting that pre-existing medical conditions and hospitalization during acute COVID-19 are risk factors for developing PCC [2,51]. However, it is important to note that existing evidence is sparse, collected from a wide variety of settings and study designs, which hinders direct comparisons and reduces the overall interpretability of findings. Besides, research has also demonstrated that PCC can occur in people who were previously healthy and experienced a mild COVID-19 infection, with one third of the individuals with PCC reporting no prior health conditions [52]. Our findings thus further highlight the heterogenous nature of PCC, suggesting that it can manifest across a broad spectrum of baseline health profiles, not limited to those with significant comorbidity burden.

A lower MIP and 6MWD %predicted were observed in the individuals with PCC compared to those without, whereas no differences were found between groups for the other measures within the muscle strength and physical capability dimensions. Specifically, mean MIP was approximately 12% lower in the PCC group. The clinical significance of this finding is uncertain, as both group means remained within the expected normal range. Similarly, the PCC group demonstrated a lower 6MWD %predicted as compared to the non-PCC group, consistent with prior research at two years follow-up [20]. Notably, 6MWD %predicted values were below the commonly used threshold of 80% predicted in both groups, indicating a degree of functional impairment irrespective of PCC status [30]. Furthermore, the absolute difference in 6MWD was modest (24 m), which was not statistically significant and did not exceed the minimally clinically important difference as established in other patient populations [53].

**Fig 6:**
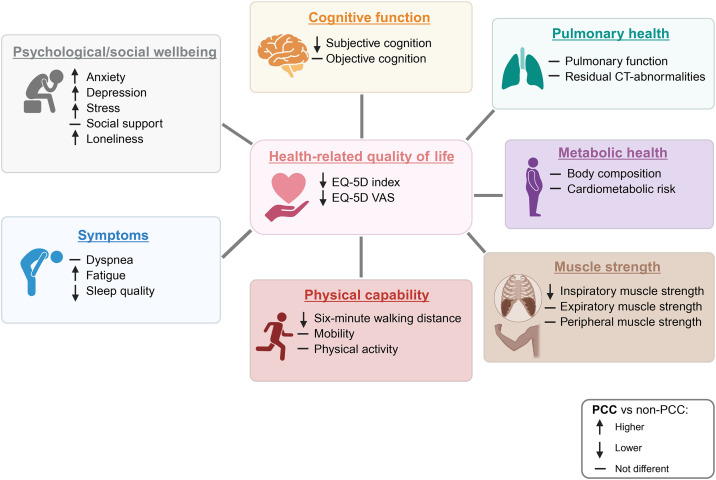
Multidimensional health outcomes in the individuals with and without self-reported PCC (n = 87/52). **Note:** Abbreviations: CT, computed tomography; EQ-5D, EuroQol five-dimensions; PCC, post COVID-19 condition. Reprinted from [Biorender] under a CC BY license, with permission from [Debbie Gach], original copyright [2026].

A comprehensive and multidimensional evaluation of long-term health outcomes has been conducted in several studies, primarily including hospitalized patients [19,20]. These studies consistently reported significant subjective health burden, despite largely resolved functional objective impairments two years post-infection [19,20], which aligns with our findings, as visually summarized in Fig 6. The dissociation between physiological recovery and patient-reported wellbeing underscores the complexity of PCC. Furthermore, the discrepancy between objective functional assessments and patients perceived health burden, may arise from the fact that self-reported measures capture a broader range of sensations, influenced by individual interpretations and symptom experience, while objective testing methods solely focus on specific functional domains which may remain unutilized in daily activities. Nonetheless, the subjective experience of health problems is crucial as it directly impacts daily functioning and overall health status [15,20], highlighting the need for further research into the biological aetiology of these persistent subjective symptoms using more advanced and in-depth methodologies. Moreover, given the considerable heterogeneity in subjective symptomatology, these findings reinforce the urgency for a multifaceted, patient-tailored approach, as emphasized in current clinical guidelines [54,55]. Specifically, the importance of a multidimensional health assessment of individuals with PCC, as impairments may span physical, psychological, and functional domains. Subsequently, the findings support the need for individualized and interdisciplinary rehabilitation approaches that address the heterogenous nature of PCC, rather than focusing on a single domain of health.

Some limitations of this study deserve discussion. The inclusion of non-hospitalized patients with persisting symptoms may have introduced selection bias, potentially leading to some overestimation of complaints. Additionally, the absence of pre COVID-19 multidimensional health outcome assessment of the study group limits our ability to establish any causality. Due to the relatively limited sample sizes in both groups, statistical power was insufficient to correct for potential confounders (i.e., comorbidity burden and hospitalization status), which represent a limitation in the current study. Hence, results are based on unadjusted comparisons, and no causality can be established from these results. The longer follow-up duration of more than one year after infection may have increased the risk of recall bias. Nevertheless, the longer follow-up duration allowed for the assessment of more persistent and potentially chronic health impairments. PCC in our cohort was self-reported rather than diagnosed through expert clinical assessment and follow-up calls to confirm self-reported PCC occurred at a mean interval of 13 ± 5.7 months after the study visit, which may have introduced some recall bias. Nevertheless, current diagnosis criteria for PCC have not yet been standardized and still rely solely on symptom reporting lacking a validated biomarker for accurate diagnosis. Notably, this study is among the few that have documented the presence of PCC, providing insights into its burden.

In conclusion, this study demonstrates that individuals with self-reported PCC (hospitalized and non-hospitalized) reported a lower HRQoL as compared to individuals without self-reported PCC approximately two years after infection. The PCC group also more frequently reported fatigue, depression, stress, anxiety and loneliness, and lower subjective cognitive functioning and sleep quality than the non-PCC group. These self-reported symptoms were only partially supported by the objectively assessed health dimensions, with lower inspiratory muscle strength and exercise capacity observed in the PCC group, while other functional measures showed no differences.These findings indicate a discordance between subjective health burden and currently available objective assessments in individuals with PCC. This highlights the need for a more patient-centred approach in post COVID care, integrating subjective assessments alongside objective measurements to better address the multifaceted impact of the condition, subsequently guiding the development of tailored treatment approaches for PCC management in the future.

## Supporting information

S1 Table Correlations between health-related quality of life and multidimensional health dimensions in total cohort (n = 139). (DOCX)

S1 Fig Symptom impairments in the individuals with and without self-reported PCC (n = 87/52). **Note:** A: CIS impairments stratified by self-reported PCC. B: PSQI impairments stratified by self-reported PCC. Data are shown as n (%) ±SD. *Significantly different between individuals with and without self-reported PCC, *p* < 0.05, **p < 0.01, ***p < 0.001. Abbreviations: CIS, Checklist Individual Strength; PCC, post COVID-19 condition; PSQI, Pittsburgh Sleep Quality Index.
(DOCX)

S2 Fig Psychological impairments in the individuals with and without self-reported PCC (n = 87/52). **Note:** A: HADS anxiety impairments stratified by self-reported PCC. B: HADS depression impairments stratified by self-reported PCC. Data are shown as n (%) ±SD. *Significantly different between individuals with and without self-reported PCC, *p* < 0.05, **p < 0.01, ***p < 0.001. Abbreviations: HADS, Hospital Anxiety and Depression Scale; PCC, post COVID-19 condition.
(DOCX)

S3 Fig Cognitive impairments in the individuals with and without self-reported PCC (n = 87/52). **Note:** A: CFQ impairments stratified by self-reported PCC. B: MoCA impairments stratified by PCC. Data are shown as n (%) ±SD. *Significantly different between individuals with and without self-reported PCC, *p* < 0.05, **p < 0.01, ***p < 0.001. Abbreviations: CFQ, Cognitive Failure Questionnaire; MoCA, Montreal Cognitive Assessment Tool; PCC, post COVID-19 condition.
(DOCX)

S1 Data Dataset PCH study V2. (XLSX)
