# Supplementary material for: Long-term multidimensional health status of individuals with and without post COVID-19 condition: A cross-sectional study
Source: PLoS One. 2026 Jul 7;21(7):e0352332. doi: 10.1371/journal.pone.0352332 (PMC13340848; doi:10.1371/journal.pone.0352332)
Supplement: S1 Table — (DOCX) [file pone.0352332.s001.docx]

**Table S1: Correlations between health-related quality of life and multidimensional health dimensions in total cohort (n=139).**

|  | **Pearson correlation with EQ-5D score** | ***p*-value** |
| --- | --- | --- |
| **Pulmonary health** |  |  |
| Pulmonary function |  |  |
| FEV_1_ in %pred | 0.127 | 0.150 |
| FVC in % pred | 0.198 | **0.024** |
| FEV_1_/FVC ratio in L | -0.165 | 0.062 |
| DLCO in %pred | -0.161 | 0.059 |
| TLC in %pred | -0.023 | 0.789 |
| **Metabolic health** |  |  |
| Body composition |  |  |
| BMI in kg/m^2^ | -0.145 | 0.092 |
| FFMI in kg/m^2^ | -0.010 | 0.910 |
| SMI in kg/m^2^ | -0.025 | 0.776 |
| BMD in g/cm^2^ | 0.018 | 0.836 |
| Cardiometabolic risk |  |  |
| Glucose in mmol/L | 0.075 | 0.386 |
| LDL cholesterol in mmol/L | -0.132 | 0.128 |
| HDL cholesterol in mmol/L | -0.017 | 0.842 |
| Triglycerides in mmol/L | -0.026 | 0.760 |
| Waist circumference in cm | -0.002 | 0.984 |
| Systolic blood pressure in mm Hg | 0.098 | 0.251 |
| Diastolic blood pressure in mm Hg | 0.019 | 0.828 |
| **Muscle strength** |  |  |
| Respiratory muscle strength |  |  |
| MIP in %pred | 0.206 | **0.015** |
| MEP in %pred | 0.319 | **<0.001** |
| Upper extremity strength |  |  |
| Max. HGS dominant in kg | 0.326 | **<0.001** |
| Lower extremity strength |  |  |
| Peak torque in Nm | 0.241 | **0.004** |
| **Physical capability** |  |  |
| Mobility |  |  |
| SPPB score | 0.284 | **0.001** |
| Cardiorespiratory fitness |  |  |
| 6MWD in m | 0.330 | **<0.001** |
| 6MWD in %pred | 0.402 | **<0.001** |
| Physical activity level |  |  |
| Total step count per day | 0.169 | **0.049** |
| Activity Score in MET.h | 0.176 | **0.040** |
| **Symptoms** |  |  |
| mMRC score | -0.468 | **<0.001** |
| CIS-fatigue score | -0.512 | **<0.001** |
| PSQI score | -0.437 | **<0.001** |
| **Psychological- and social wellbeing** |  |  |
| HADS anxiety score | -0.481 | **<0.001** |
| HADS depression score | -0.551 | **<0.001** |
| PSS score | -0.492 | **<0.001** |
| MSPSS score | 0.451 | **<0.001** |
| LS score | -0.446 | **<0.001** |
| **Cognitive function** |  |  |
| CFQ score | -0.392 | **<0.001** |
| MoCA score | 0.241 | **0.004** |

**Note:** Pearson correlation coefficients (*r*) are shown. Bold indicates a significant correlation between EQ-5D score and respective measure of multidimensional health dimension, *p*<0.05. Abbreviations: BMD, bone mineral density; BMI, body mass index; CFQ, Cognitive Failure Questionnaire; CIS, Checklist Individual Strength; DLCO, diffusion capacity of the lungs for carbon monoxide; FEV_1_, forced expiratory volume in one second; FFMI, fat free mass index; FVC, forced vital capacity; HADS, Hospital Anxiety and Depression Scale; HDL, high-density lipoprotein; HGS, handgrip strength; LDL, low-density lipoprotein; LS, Loneliness Scale; MEP, maximal expiratory pressure; MIP, maximal inspiratory pressure; MoCA, Montreal Cognitive Assessment; mMRC, modified Medical Research Council; MSPSS, Multidimensional Scale of Perceived Social Support; 6MWD, 6-minute walking distance; PSQI, Pittsburgh Sleep Quality Index; PSS, Perceived Stress Scale; SMI, skeletal muscle mass index; SPPB, Short Physical Performance Battery; TLC, total lung capacity.
