# Supplementary material for: Long-term multidimensional health status of individuals with and without post COVID-19 condition: A cross-sectional study
Source: PLoS One. 2026 Jul 7;21(7):e0352332. doi: 10.1371/journal.pone.0352332 (PMC13340848; doi:10.1371/journal.pone.0352332)
Supplement: S2 Fig — Note: A: HADS anxiety impairments stratified by self-reported PCC. B: HADS depression impairments stratified by self-reported PCC. Data are shown as n (%) ±SD. *Significantly different between individuals with and without self-reported PCC, p < 0.05, **p < 0.01, ***p < 0.001. Abbreviations: HADS, Hospital Anxiety and Depression Scale; PCC, post COVID-19 condition. (DOCX) [file pone.0352332.s003.docx]

**
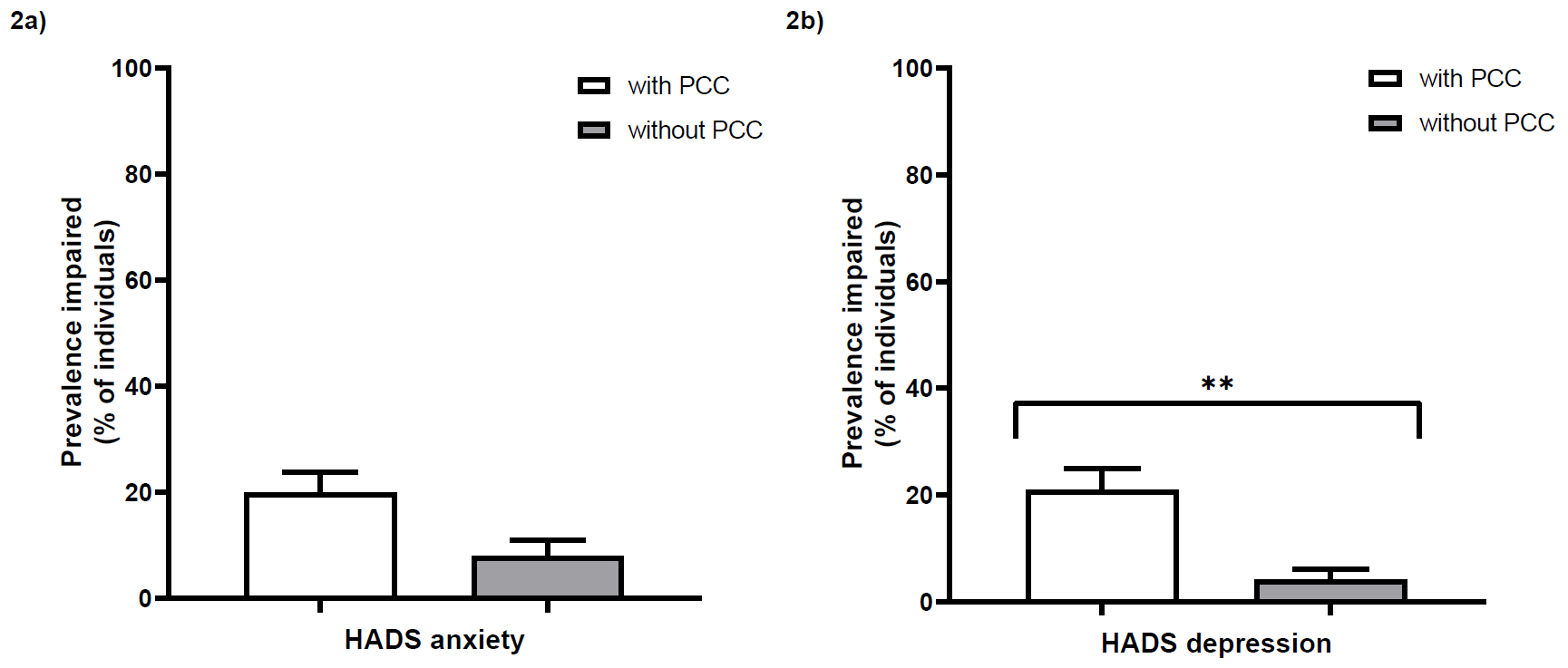
**

**Figure S2: Psychological impairments in the individuals with and without self-reported PCC (n=87/52).**

**Note:** A: HADS anxiety impairments stratified by self-reported PCC. B: HADS depression impairments stratified by self-reported PCC. Data are shown as n (%) ±SD. *Significantly different between individuals with and without self-reported PCC, *p*<0.05, ***p*<0.01, ****p*<0.001. Abbreviations: HADS, Hospital Anxiety and Depression Scale; PCC, post COVID-19 condition.
