# Supplementary material for: Long-term multidimensional health status of individuals with and without post COVID-19 condition: A cross-sectional study
Source: PLoS One. 2026 Jul 7;21(7):e0352332. doi: 10.1371/journal.pone.0352332 (PMC13340848; doi:10.1371/journal.pone.0352332)
Supplement: Attachment — Submitted filename: Reviewer comments.pdf [file pone.0352332.s006.pdf]

Reviewer

It is an honor to review the manuscript entitled **"Long-term multidimensional health status of individuals with and without post-COVID-19 condition: a cross-sectional study" (PONE-D-25-63012)**. I have carefully reviewed this manuscript and found that it addresses high-quality, clinically relevant issues, employs strong methodology, and conveys an important message. It presents a well-designed, comprehensive multidimensional assessment of long-term health outcomes in individuals with and without self-reported post-COVID-19 condition (PCC) at ≥1 year after SARS-CoV-2 infection. The topic is timely, clinically relevant, and aligned with PLOS ONE's scope. The study's strengths include the breadth of objective and subjective assessments, inclusion of both hospitalized and non-hospitalized patients, and the direct comparison between PCC and non-PCC groups. However, minor issues should be addressed to strengthen the manuscript, particularly regarding the PCC definition, potential confounding, causal interpretation, and writing style, in accordance with PLOS ONE's guidelines. After this revision, I support accepting this manuscript in PLOS ONE.

Manor's suggestion and revision

1. As I understand it, PLOS ONE's guidelines do not require subtitles in the abstract. Instead, the abstract should be a concise summary of fewer than 300 words that covers the study's objective, methods (briefly), key results, and significance, without citations or excessive abbreviations.

2. I have a major concern about this. **Please read this carefully.** It may help strengthen your manuscripts. Post-COVID-19 condition (PCC) was defined solely on the basis of retrospective self-report during a follow-up phone call, using three yes/no questions. This approach may introduce recall bias, subjectivity, and potential circularity between symptom reporting and outcome measures. This limitation should be explicitly acknowledged in both the Methods and Discussion sections. The authors should also clarify why a standardized PCC definition, such as WHO-based symptom criteria or duration thresholds, was not applied. Where possible, sensitivity analyses using symptom burden thresholds (e.g., fatigue, dyspnea, or cognitive complaints) could strengthen the findings, and causal language should be carefully revised to reflect associations rather than causation.

3. The author has shown that there were significant baseline differences between the PCC and non-PCC groups, including the Charlson Comorbidity Index, smoking status, hospitalization rates, and COVID-19 wave distribution, which may confound comparisons of health-related quality of life and physical performance. To address this, adjusted analyses, such as multivariable regression controlling for age, sex, comorbidity burden, hospitalization status, and time since infection, are recommended. At a minimum, please clearly state that the results are based on unadjusted comparisons and interpret the findings with appropriate caution.

4. In the abstract, at the end of Lines 56-57, the author states that **this suggests current objective testing methods could not adequately reflect patients' perceived health burden in PCC**, a claim that is not fully supported by the cross-sectional study design. The findings indicate a discordance between subjective health burden and currently available objective assessments in individuals with post-COVID-19 condition.
