# Supplementary material for: Long-term multidimensional health status of individuals with and without post COVID-19 condition: A cross-sectional study
Source: PLoS One. 2026 Jul 7;21(7):e0352332. doi: 10.1371/journal.pone.0352332 (PMC13340848; doi:10.1371/journal.pone.0352332)
Supplement: Attachment — Submitted filename: Reviewer 3 comments.pdf [file pone.0352332.s008.pdf]

Reviewer 3.

It is wonderful to receive the revised manuscript titled **"Long-term multidimensional health status of individuals with and without post-COVID-19 condition: a cross-sectional study" (PONE-D-25-63012R1).** The authors have appropriately addressed several important concerns, including PCC, potential confounding, causal interpretation, and clarity of writing. Thus, I recommend acceptance after minor revision. Overall, the manuscript is scientifically solid, clinically relevant, and methodologically transparent. However, a few relatively minor issues could be addressed before final acceptance. The data availability statement ("Datasets are available upon request") may not fully comply with PLOS ONE's strict data-sharing policy and may require clarification or repository deposition. In addition, the study still relies on retrospective self-reported PCC classification, and the comparisons remain unadjusted for baseline differences such as hospitalization and comorbidity burden. Nevertheless, the authors now openly acknowledge these limitations, which substantially strengthen the manuscript.
