# Supplementary material for: Long-term multidimensional health status of individuals with and without post COVID-19 condition: A cross-sectional study
Source: PLoS One. 2026 Jul 7;21(7):e0352332. doi: 10.1371/journal.pone.0352332 (PMC13340848; doi:10.1371/journal.pone.0352332)
Supplement: Attachment — Submitted filename: Response to reviewers.docx [file pone.0352332.s009.docx]

Maastricht, The Netherlands

March 30, 2026

Dear dr. She,

Thank you for your e-mail on the 13^th^ of February regarding our submission of the manuscript entitled “Long-term multidimensional health status of individuals with and without post COVID-19 condition: a cross-sectional study” with manuscript ID: PONE-D-25-63012.

We appreciate the consideration of our manuscript by the editor and reviewers for submission to *PLOS One*. Revisions in the manuscript have been made according to the editor and reviewers’ comments to the initial manuscript. We have uploaded the revised document with track changes and the clean version. In addition, within this document, a letter has been written including our responses to the reviewers’ comments in a point-by-point manner.

Besides, the amended Funding Statement for this manuscript is as follows: This study was supported by a Research Fellowship from the European Society of Clinical Nutrition and Metabolism (ESPEN) awarded to Rosanne J.H.C.G. Beijers, and by a ZonMw grant (project number 100430040211004) awarded to Hester A. Gietema, and Annemie M.W.J. Schols funded this work. The funders had no role in study design, data collection and analysis, decision to publish, or preparation of the manuscript. There was no additional external funding received for this study.

Additionally, the amended Competing Interests Statements reads as follows: I have read the journal's policy and the authors of this manuscript have the following competing interests: Competing interests statement: J.P. van den Bergh has received research funding from POROUS (paid to institution) not related to this work and consultancy fee (paid to institution) not related to this work. There are no other relevant declarations. This does not alter our adherence to PLOS ONE policies on sharing data and materials.

We hope our revised manuscript will now be considered for publication in *PLOS One.*

Kind regards,

The authors

**Journal Requirements:**

When submitting your revision, we need you to address these additional requirements.

1. Please ensure that your manuscript meets PLOS ONE's style requirements, including those for file naming. The PLOS ONE style templates can be found at

[https://journals.plos.org/plosone/s/file?id=wjVg/PLOSOne_formatting_sample_main_body.pdf](https://track.editorialmanager.com/CL0/https:%2F%2Fjournals.plos.org%2Fplosone%2Fs%2Ffile%3Fid=wjVg%2FPLOSOne_formatting_sample_main_body.pdf/1/010f019c559670b2-7e55591f-ff00-40b0-9be8-28d40f6f6858-000000/-mZVHm_PfsVN70o5DzUP504MN42xHIIkv-WVtSHlm04=250) [https://journals.plos.org/plosone/s/file?id=ba62/PLOSOne_formatting_sample_title_authors_affiliations.pdf](https://track.editorialmanager.com/CL0/https:%2F%2Fjournals.plos.org%2Fplosone%2Fs%2Ffile%3Fid=ba62%2FPLOSOne_formatting_sample_title_authors_affiliations.pdf/1/010f019c559670b2-7e55591f-ff00-40b0-9be8-28d40f6f6858-000000/WnlLPEK0GbpLxe3xvcfJmlcb4OuEVhdU3Ecb1Ge_E6c=250)

*We have double-checked the journals requirements and adjusted the manuscript accordingly.*

2. Thank you for stating in your Funding Statement:

[Funding statement: This study was supported by a Research Fellowship from the European Society of Clinical Nutrition and Metabolism (ESPEN) awarded to Rosanne J.H.C.G. Beijers, and by a ZonMw grant (project number 100430040211004) awarded to Hester A. Gietema, and Annemie M.W.J. Schols funded this work. The funders had no role in study design, data collection and analysis, decision to publish, or preparation of the manuscript.].

Please provide an amended statement that declares *all* the funding or sources of support (whether external or internal to your organization) received during this study, as detailed online in our guide for authors at <http://journals.plos.org/plosone/s/submit-now>.  Please also include the statement “There was no additional external funding received for this study.” in your updated Funding Statement.

Please include your amended Funding Statement within your cover letter. We will change the online submission form on your behalf.

*We have added the amended Funding Statement within the cover letter at the start of this document.*

3. Thank you for stating the following in your manuscript:

[This study was supported by a Research Fellowship from the European Society of Clinical Nutrition and Metabolism (ESPEN) awarded to Rosanne J.H.C.G. Beijers, and by a ZonMw grant (project number 100430040211004) awarded to Hester A. Gietema, and Annemie M.W.J. Schols funded this work. The funders had no role in study design, data collection and analysis, decision to publish, or preparation of the manuscript.]

We note that you have provided funding information that is currently declared in your Funding Statement. However, funding information should not appear in the Acknowledgments section or other areas of your manuscript. We will only publish funding information present in the Funding Statement section of the online submission form.

Please remove any funding-related text from the manuscript and let us know how you would like to update your Funding Statement. Currently, your Funding Statement reads as follows:

[Funding statement: This study was supported by a Research Fellowship from the European Society of Clinical Nutrition and Metabolism (ESPEN) awarded to Rosanne J.H.C.G. Beijers, and by a ZonMw grant (project number 100430040211004) awarded to Hester A. Gietema, and Annemie M.W.J. Schols funded this work. The funders had no role in study design, data collection and analysis, decision to publish, or preparation of the manuscript.]

Please include your amended statements within your cover letter; we will change the online submission form on your behalf.

*We have removed the Funding Statement in the manuscript and added the amended Funding Statement within the cover letter at the start of this document.*

4. Thank you for stating the following in the Competing Interests section:

[I have read the journal's policy and the authors of this manuscript have the following competing interests: J. van den Bergh has research funding from POROUS, and consultancy from UCB and AMGEN, which was not related to this work. No competing interests exist for the other authors.].

We note that you received funding from commercial sources: POROUS, UCB, and AMGEN.

Please provide an amended Competing Interests Statement that explicitly states this commercial funder, along with any other relevant declarations relating to employment, consultancy, patents, products in development, marketed products, etc.

Within this Competing Interests Statement, please confirm that this does not alter your adherence to all PLOS ONE policies on sharing data and materials by including the following statement: "This does not alter our adherence to PLOS ONE policies on sharing data and materials.” (as detailed online in our guide for authors <http://journals.plos.org/plosone/s/competing-interests>).  If there are restrictions on sharing of data and/or materials, please state these. Please note that we cannot proceed with consideration of your article until this information has been declared.

Please include your amended Competing Interests Statement within your cover letter. We will change the online submission form on your behalf.

*We have added the amended Competing Interests Statement within the cover letter at the beginning of this document.*

5. One of the noted authors is a group or consortium [COVID CLIMATE Consortium]. In addition to naming the author group, please list the individual authors and affiliations within this group in the acknowledgments section of your manuscript. Please also indicate clearly a lead author for this group along with a contact email address.

*We have listed the individual authors in the acknowledgement section of this manuscript. Additionally, A.M.W.J. Schols was the lead author for this group (*[*a.schols@maastrichtuniversity.nl*](mailto:a.schols@maastrichtuniversity.nl)*).*

6. We note that Figures 2 and 6 in your submission contain images which may be copyrighted. All PLOS content is published under the Creative Commons Attribution License (CC BY 4.0), which means that the manuscript, images, and Supporting Information files will be freely available online, and any third party is permitted to access, download, copy, distribute, and use these materials in any way, even commercially, with proper attribution. For more information, see our copyright guidelines: <http://journals.plos.org/plosone/s/licenses-and-copyright>.

We require you to either (1) present written permission from the copyright holder to publish these figures specifically under the CC BY 4.0 license, or (2) remove the figures from your submission:

1. You may seek permission from the original copyright holder of Figures 2 and 6 to publish the content specifically under the CC BY 4.0 license.

We recommend that you contact the original copyright holder with the Content Permission Form (<http://journals.plos.org/plosone/s/file?id=7c09/content-permission-form.pdf>) and the following text:

“I request permission for the open-access journal PLOS ONE to publish XXX under the Creative Commons Attribution License (CCAL) CC BY 4.0 (<http://creativecommons.org/licenses/by/4.0/>). Please be aware that this license allows unrestricted use and distribution, even commercially, by third parties. Please reply and provide explicit written permission to publish XXX under a CC BY license and complete the attached form.”

Please upload the completed Content Permission Form or other proof of granted permissions as an "Other" file with your submission.

In the figure caption of the copyrighted figure, please include the following text: “Reprinted from [ref] under a CC BY license, with permission from [name of publisher], original copyright [original copyright year].”

2. If you are unable to obtain permission from the original copyright holder to publish these figures under the CC BY 4.0 license or if the copyright holder’s requirements are incompatible with the CC BY 4.0 license, please either i) remove the figure or ii) supply a replacement figure that complies with the CC BY 4.0 license. Please check copyright information on all replacement figures and update the figure caption with source information. If applicable, please specify in the figure caption text when a figure is similar but not identical to the original image and is therefore for illustrative purposes only.

*We have uploaded the completed Content Permission Form as “Other” file with the resubmission of our manuscript.*

7. Please include captions for your Supporting Information files at the end of your manuscript, and update any in-text citations to match accordingly. Please see our Supporting Information guidelines for more information: [http://journals.plos.org/plosone/s/supporting-information](http://track.editorialmanager.com/CL0/http:%2F%2Fjournals.plos.org%2Fplosone%2Fs%2Fsupporting-information/1/010f019c559670b2-7e55591f-ff00-40b0-9be8-28d40f6f6858-000000/GY5TM3N8C1fGbHuRslPXcgEVFSTzzSlPBacPdCHtKqI=250).

*We have adjusted the captions of the Supporting Information files and matched the in-text citations accordingly as requested by PLOS One’s submission guidelines.*

If the reviewer comments include a recommendation to cite specific previously published works, please review and evaluate these publications to determine whether they are relevant and should be cited. There is no requirement to cite these works unless the editor has indicated otherwise. 

Please review your reference list to ensure that it is complete and correct. If you have cited papers that have been retracted, please include the rationale for doing so in the manuscript text, or remove these references and replace them with relevant current references. Any changes to the reference list should be mentioned in the rebuttal letter that accompanies your revised manuscript. If you need to cite a retracted article, indicate the article’s retracted status in the References list and also include a citation and full reference for the retraction notice.
*We have double-checked the reference list and made adjustments as appropriate.*

Reviewers' comments:

Reviewer's Responses to Questions

**Comments to the Author**

1. Is the manuscript technically sound, and do the data support the conclusions?

The manuscript must describe a technically sound piece of scientific research with data that supports the conclusions. Experiments must have been conducted rigorously, with appropriate controls, replication, and sample sizes. The conclusions must be drawn appropriately based on the data presented.

Reviewer #1: Yes

Reviewer #2: Yes

Reviewer #3: Yes

Reviewer #4: Yes

2. Has the statistical analysis been performed appropriately and rigorously?

Reviewer #1: Yes

Reviewer #2: Yes

Reviewer #3: Yes

Reviewer #4: Yes

3. Have the authors made all data underlying the findings in their manuscript fully available?

The [PLOS Data policy](http://track.editorialmanager.com/CL0/http:%2F%2Fwww.plosone.org%2Fstatic%2Fpolicies.action%23sharing/1/010f019c559670b2-7e55591f-ff00-40b0-9be8-28d40f6f6858-000000/9xvwcCN_U7GVGDD6kTEGYJM10n93E8R4F6i05jjrFJw=250) requires authors to make all data underlying the findings described in their manuscript fully available without restriction, with rare exception (please refer to the Data Availability Statement in the manuscript PDF file). The data should be provided as part of the manuscript or its supporting information, or deposited to a public repository. For example, in addition to summary statistics, the data points behind means, medians and variance measures should be available. If there are restrictions on publicly sharing data—e.g. participant privacy or use of data from a third party—those must be specified.

Reviewer #1: Yes

Reviewer #2: Yes

Reviewer #3: Yes

Reviewer #4: Yes

4. Is the manuscript presented in an intelligible fashion and written in standard English?

PLOS ONE does not copyedit accepted manuscripts, so the language in submitted articles must be clear, correct, and unambiguous. Any typographical or grammatical errors should be corrected at revision, so please note any specific errors here.

Reviewer #1: Yes

Reviewer #2: Yes

Reviewer #3: Yes

Reviewer #4: Yes

5. Review Comments to the Author

Please use the space provided to explain your answers to the questions above. You may also include additional comments for the author, including concerns about dual publication, research ethics, or publication ethics. (Please upload your review as an attachment if it exceeds 20,000 characters)

Reviewer #1: This study presents a technically sound, well-written analysis that demonstrates differences in COVID-19 severity. It is an exciting article to publish. The title is Long-term multidimensional health status of individuals with and without post COVID-19 condition: a cross-sectional study, and the ID number is (PONE-D-25-63012). The authors should run the manuscript through Grammarly to ensure spelling and grammar are correct before publishing the article.

*We thank the reviewer for their time and efforts to review our manuscript and for their valuable comments. The manuscript has been carefully reviewed using grammar-checking tools for spelling and grammatical accuracy, and we have made minor revisions throughout to improve clarity and readability in accordance with PLOS ONE standards.*

Reviewer #2: Major comments

1. The follow-up interval in this study introduces a potential risk of recall bias among participants. The rationale for selecting a minimum follow-up duration of one year should be more clearly justified, and the reasons for not applying the WHO symptom-duration criteria for PCC (minimum three months) at the time of assessment should be explicitly explained.

*We thank the reviewer for their time and effort to critically evaluate our manuscript and for this important comment. We acknowledge that the relatively long follow-up interval may introduce a risk of recall bias, particularly for self-reported outcomes. This limitation has now been more explicitly addressed in the Discussion section of the revised manuscript (Page 24-25, Lines 434-437).*

*At the time of the study design (early in the COVID-19 pandemic), post COVID-19 condition (PCC) was not yet defined, and the WHO symptom-duration criteria (≥3 months) had not been established. Therefore, these criteria could not be applied when designing the study protocol.*

*The choice for a follow-up duration of ≥1 year was made to ensure that potential long-term health consequences were adequately captured. This decision was informed by previous evidence from infections with related coronaviruses, such as Severe Acute Respiratory Syndrome, where persistent impairments beyond one year have been reported (doi:* [*10.7861/clinmed.2020-0204*](https://doi.org/10.7861/clinmed.2020-0204) */ doi: 10.1111/j.1440-1843.2010.01720.x). Given the uncertainty at that time regarding the trajectory and persistence of COVID-19-related sequelae, a longer follow-up period was considered most appropriate. This rationale has now been added to the Method section in our manuscript (Page 7, Lines 118-122 and Page 14, Lines 217-220).*

*In addition, we would like to emphasize that the ≥1 year follow-up can also be considered a strength of the study, as it allows for the assessment of more persistent and potentially chronic health impairments, rather than transient symptoms that may be resolved within the first months after infection. This provides complementary insights into studies using shorter follow-up intervals based on the later-established WHO PCC definition.*

2. In addition, the delayed, self-reported classification of PCC may introduce result-driven classification bias due to increased recall bias. This temporal gap between the multidimensional health assessment and PCC classification may affect the internal validity of group comparisons and correlation analyses. The authors are encouraged to clarify whether symptom status reported at follow-up accurately reflected participants’ health status at the time of objective testing.

*We agree with the reviewer that the delayed, self-reported classification of PCC may have introduced recall bias, which is now explicitly acknowledged in the limitation section of the revised manuscript (Page 24-25, Lines 434-437).*

*However, as described in the Methods section (“Follow-up call”) (Page 14, Lines 214-217), participants were specifically asked whether they experienced symptoms related to COVID-19 at the time of the study visit that they attributed to PCC. This approach was intended to reflect their health status at the time of objective testing as accurately as possible, although some degree of recall bias cannot be excluded.*

3. Lack of adjustment for baseline group differences (e.g., CCI, hospitalization) should be noted as a limitation

*We agree with the reviewer that the lack of adjustment for baseline differences, such as comorbidity burden (CCI) and hospitalization status, represents a limitation of the present study. Due to the relatively limited sample sizes in both groups, statistical power was insufficient to reliably adjust for these potential confounders without increasing the risk of model overfitting. We have now explicitly acknowledged this limitation in the revised manuscript (Discussion section Page 24, Lines 430-434).*

Minor Comments
1. Add a short note on clinical and rehabilitation implications.

*We thank the reviewer for this helpful suggestion. We have elaborated on the clinical and rehabilitation implications in the Discussion section (Page 24, Lines 415-419).*

2. Correct minor formatting and ensure consistent terminology and supplementary figure referencing.

*We have double-checked the formatting guidelines of PLOS One and made the correct changes accordingly to ensure proper formatting and consistent terminology throughout the whole manuscript.*

Reviewer #3: Reviewer

It is an honor to review the manuscript entitled "Long-term multidimensional health status of individuals with and without post-COVID-19 condition: a cross-sectional study” (PONE-D-25-63012). I have carefully reviewed this manuscript and found that it addresses high-quality, clinically relevant issues, employs strong methodology, and conveys an important message. It presents a well-designed, comprehensive multidimensional assessment of long-term health outcomes in individuals with and without self-reported post-COVID-19 condition (PCC) at ≥1 year after SARS-CoV-2 infection. The topic is timely, clinically relevant, and aligned with PLOS ONE’s scope. The study’s strengths include the breadth of objective and subjective assessments, inclusion of both hospitalized and non-hospitalized patients, and the direct comparison between PCC and non-PCC groups. However, minor issues should be addressed to strengthen the manuscript, particularly regarding the PCC definition, potential confounding, causal interpretation, and writing style, in accordance with PLOS ONE’s guidelines. After this revision, I support accepting this manuscript in PLOS ONE.

*We thank the reviewer for their thorough and constructive evaluation of our manuscript, as well as for their positive appraisal of its clinical relevance, methodological rigor, and overall contribution. We appreciate the reviewer’s insightful comments regarding the definition of PCC, potential confounding, causal interpretation, and writing clarity, and have addressed these points in detail below.*

Major’s suggestion and revision

1. As I understand it, PLOS ONE’s guidelines do not require subtitles in the abstract. Instead, the abstract should be a concise summary of fewer than 300 words that covers the study's objective, methods (briefly), key results, and significance, without citations or excessive abbreviations.

*We agree with the reviewer that, according to PLOS ONE guidelines, the abstract does not require subtitles but should be a concise summary of fewer than 300 words covering the study’s objective, methods, key results, and significance. We have therefore revised the abstract, accordingly, removing the subtitles and ensuring compliance with these requirements (Page 3-4, Lines 32-57).*

2. I have a major concern about this. Please read this carefully. It may help strengthen your manuscripts. Post-COVID-19 condition (PCC) was defined solely on the basis of retrospective self-report during a follow-up phone call, using three yes/no questions. This approach may introduce recall bias, subjectivity, and potential circularity between symptom reporting and outcome measures. This limitation should be explicitly acknowledged in both the Methods and Discussion sections. The authors should also clarify why a standardized PCC definition, such as WHO-based symptom criteria or duration thresholds, was not applied. Where possible, sensitivity analyses using symptom burden thresholds (e.g., fatigue, dyspnea, or cognitive complaints) could strengthen the findings, and causal language should be carefully revised to reflect associations rather than causation.

*We thank the reviewer for this important and insightful comment. We acknowledge that defining PCC based on retrospective self-report during follow-up may introduce recall bias and subjectivity, as well as potential overlap between symptom reporting and outcome measures. This limitation has now been explicitly addressed in Discussion section (Page 24-25, Lines 434–443). However, as described in the Methods section (“Follow-up call”) Page 14, Lines 214-217, participants were specifically asked whether they experienced symptoms related to COVID-19 at the time of the study visit that they attributed to PCC. This approach was intended to reflect their health status at the time of objective testing as accurately as possible, although some degree of recall bias cannot be excluded.*

*We have also clarified our rationale for not applying a standardized definition such as the WHO-based criteria. Specifically, PCC classification in our study was based on self-reported persistent symptoms during follow-up, which reflects real-world clinical reporting but may differ from formal diagnostic frameworks, which were not yet available at the time the study was designed and could therefore not be applied in the present study. This has now been explained in the Methods section (Page 7, Lines 118-122 and Page 14, Lines 217-220).*

*We agree that sensitivity analyses based on symptom burden thresholds could further strengthen the findings. However, due to limited statistical power for such subgroup analyses, we were unable to perform these reliably. This limitation has now been explicitly mentioned in the Discussion section of our manuscript (Page 24, Lines 430-433).*

*Finally, we have carefully revised the manuscript to avoid causal language and to consistently reflect associative interpretations of the results.*

3. The author has shown that there were significant baseline differences between the PCC and non-PCC groups, including the Charlson Comorbidity Index, smoking status, hospitalization rates, and COVID-19 wave distribution, which may confound comparisons of health-related quality of life and physical performance. To address this, adjusted analyses, such as multivariable regression controlling for age, sex, comorbidity burden, hospitalization status, and time since infection, are recommended. At a minimum, please clearly state that the results are based on unadjusted comparisons and interpret the findings with appropriate caution.

*We agree with the reviewer that the observed baseline differences between the PCC and non-PCC groups may confound comparisons of health outcomes. Due to the relatively limited sample size in both groups, performing multivariable analyses with multiple covariates would increase the risk of model overfitting and yield unstable estimates.*

*We have therefore not included adjusted analyses in the current manuscript but have explicitly acknowledged this as a limitation in the revised manuscript and clarified that all results are based on unadjusted comparisons (Discussion section, Page 24, Lines 430-433). In addition, we have revised the interpretation of our findings to reflect this limitation and to emphasize cautious, associative interpretation.*

4. In the abstract, at the end of Lines 56-57, the author states that this suggests current objective testing methods could not adequately reflect patients’ perceived health burden in PCC, a claim that is not fully supported by the cross-sectional study design. The findings indicate a discordance between subjective health burden and currently available objective assessments in individuals with post-COVID-19 condition.

*We thank the reviewer for this important comment. We agree that the original wording in the abstract may have implied a causal interpretation that is not supported by the cross-sectional design. We have therefore revised this statement to more accurately reflect our findings. The sentence now reads: “The findings indicate a discordance between subjective health burden and currently available objective assessments in individuals with post-COVID-19 condition” (Page 3-4, Lines 56-57).*

Reviewer #4: Authors present a cross-sectional assessment of long-term, multidimensional health status in individuals with and without self-reported post COVID-19 condition (PCC), evaluated at least one year after SARS-CoV-2 infection. The authors combine subjective and objective measures across multiple domains, including health-related quality of life, symptoms, psychological and social wellbeing, pulmonary and metabolic health, muscle strength, physical capability, and cognition. The study addresses a relevant clinical question and provides a broad overview of how perceived health burden relates to objectively measured outcomes in this population. While the overall design and structure are clear, some aspects—particularly the operational definition of PCC, the characteristics of the comparison group, and the interpretation of discrepancies between subjective and objective findings—would benefit from additional clarification. With these points addressed, I believe the manuscript would be appropriate for publication.

*We thank the reviewer for their thoughtful and constructive evaluation of our manuscript, as well as for highlighting its clinical relevance. We appreciate the reviewer’s comments regarding the operational definition of PCC, the characteristics of the comparison group, and the interpretation of discrepancies between subjective and objective outcomes. These points have been addressed and clarified in the revised manuscript, as detailed in our responses below.*

Points that would benefit from clarification:

1. PCC is defined here based on self-report, including the follow-up call. I would like some clarification on how misclassification was handled. Were alternative diagnoses actively excluded? How closely does your operational definition align with the WHO criteria in terms of symptom onset and duration (≥3 months after infection, lasting ≥2 months)? A clearer mapping of your definition to current PCC guidance would help.

*We thank the reviewer for this valuable comment. We acknowledge that PCC was based on self-reported symptoms, which may have introduced some misclassification bias. Alternative diagnoses were not actively excluded, reflecting real-world clinical reporting of persistent COVID-19–related symptoms. Regarding alignment with current guidance, our operational definition captures persistent symptoms attributed to COVID-19 at the time of follow-up; however, formal WHO criteria (symptom onset ≥3 months post-infection, lasting ≥2 months) were not yet available when the study was designed. We have clarified this mapping in the revised Methods section to provide context on how our definition relates to current PCC guidance (Page 7, Lines 118-122 and Page 14, Lines 217-220).*

2. Participants were recruited from hospitalized cohorts and from a post-COVID outpatient clinic, which may already select for individuals with ongoing complaints. I am not fully clear on how the “without PCC” group should be interpreted in this context. What were the main reasons these participants attended the assessment, and how confident can we be that this group represents a true post-infection comparator rather than a clinically selected population?

*We thank the reviewer for this important comment. The “without PCC” group consisted of individuals with a confirmed SARS-CoV-2 infection who did not report persistent symptoms attributed to PCC at follow-up. This group included both non-hospitalized individuals attending the post-COVID outpatient clinic and previously hospitalized patients whose primary reason for admission was SARS-CoV-2 infection.*

*Importantly, inclusion in this study was based on prior infection status rather than the presence of ongoing PCC-related complaints, supporting the interpretation of this group as a post-infection comparator rather than a population selected specifically for persistent symptoms.*

*Nevertheless, we acknowledge that individuals attending follow-up assessments may still represent a clinically selected population, potentially including participants with health concerns unrelated to PCC. This should be considered when interpreting the findings, as the “without PCC” group may not fully represent an asymptomatic post-infection population from the general community, but rather reflects a clinically relevant, real-world sample.*

3. An interesting finding is that individuals with PCC had fewer comorbidities and were less often hospitalized than those without PCC, which contrasts with part of the existing literature. This raises the question of potential confounding or selection effects. Were key outcomes (e.g., HRQoL, fatigue, sleep quality, MIP, 6MWD%pred) examined with adjustment for relevant variables such as age, sex, time since infection, hospitalization/ICU, CCI, smoking status, and infection wave or vaccination period? Even a brief adjusted or sensitivity analysis would make the interpretation more robust.

*We agree with the reviewer that the observed baseline differences between the PCC and non-PCC groups may confound comparisons of health outcomes. Given the relatively limited sample size in both groups, including a large number of covariates in multivariable models would increase the risk of overfitting and result in unstable estimates.*

*To address this concern while maintaining model robustness, we performed exploratory adjusted analyses using a limited set of key covariates (age, sex, and CCI). The results of these analyses are presented in rebuttal Table 1 and were broadly consistent with the unadjusted findings across the main outcomes, including HRQoL, fatigue, MIP, and 6MWD%pred.*

**Rebuttal Table 1: Association between outcome measures and PCC in multivariable logistic regression analysis corrected for sex, age and CCI.**

| **Variable** | **OR (95% CI)** | **p-value** |
| --- | --- | --- |
| 6MWD (%pred) | 0.96 (0.93-0.99) | 0.019 |
| MIP (%pred) | 0.99 (0.97-1.00) | 0.023 |
| HRQoL | 0.95 (0.92-0.98) | <0.001 |
| CIS-score | 1.03 (1.01-1.04) | <0.001 |
| PSQI-score | 1.11 (1.00-1.23) | 0.046 |

*We acknowledge that additional factors such as time since infection, hospitalization/ICU status, smoking status, and infection wave or vaccination period may also influence these outcomes. However, given the sample size constraints, these variables could not be reliably included in the models. This should be considered when interpreting the results as further described in the limitation section of our manuscript (Page 24, Lines 430-433).*

4. You report clear differences in subjective cognitive complaints (CFQ) but not in objective screening (MoCA). I wonder whether MoCA may simply lack sensitivity for the type of cognitive difficulties reported by PCC patients (e.g., attention, executive function, fatigue-related issues). Some discussion on measurement sensitivity, and on whether fatigue, sleep disturbance, or mood could be mediating subjective cognitive complaints, would help frame this discrepancy.

*We thank the reviewer for this insightful comment. We agree that the discrepancy between increased subjective cognitive complaints (CFQ) and the absence of differences in objective cognitive screening (MoCA) may, at least in part, reflect differences in measurement sensitivity.*

*The MoCA is designed as a brief screening tool for global cognitive impairment and may lack sensitivity to detect subtle or domain-specific deficits, particularly in areas such as attention, executive functioning, and cognitive fatigability, which are commonly reported by individuals with PCC. In contrast, the CFQ captures self-perceived cognitive difficulties in daily life, which may be more sensitive to these subtle and fluctuating impairments.*

*In addition, we acknowledge that subjective cognitive complaints may be influenced by other factors frequently observed in PCC, such as fatigue, sleep disturbances, and psychological distress. These factors may contribute to or mediate the perception of cognitive dysfunction, even in the absence of measurable deficits on brief objective screening instruments.*

*We have added text to the discussion to reflect these considerations and to better contextualize the observed discrepancy between subjective and objective cognitive outcomes (Page 22, Lines 362-372).*

If you address these points, the overall interpretation will be clearer.

6. PLOS authors have the option to publish the peer review history of their article ([what does this mean?](https://track.editorialmanager.com/CL0/https:%2F%2Fjournals.plos.org%2Fplosone%2Fs%2Feditorial-and-peer-review-process%23loc-peer-review-history/1/010f019c559670b2-7e55591f-ff00-40b0-9be8-28d40f6f6858-000000/qBn01Mr-JqPulAPdrhdBuwma7LNYuroDzFaXUML6vyw=250)). If published, this will include your full peer review and any attached files.

If you choose “no”, your identity will remain anonymous but your review may still be made public.
**Do you want your identity to be public for this peer review?** For information about this choice, including consent withdrawal, please see our [Privacy Policy](https://track.editorialmanager.com/CL0/https:%2F%2Fwww.plos.org%2Fprivacy-policy/1/010f019c559670b2-7e55591f-ff00-40b0-9be8-28d40f6f6858-000000/7YKI1nuF5yGYWMv38Xko5UShmqrwTYSc2Qlg7WQ8FnM=250).

Reviewer #1: No

Reviewer #2: No

Reviewer #3: No

Reviewer #4: No
