# Supplementary material for: Long-term multidimensional health status of individuals with and without post COVID-19 condition: A cross-sectional study
Source: PLoS One. 2026 Jul 7;21(7):e0352332. doi: 10.1371/journal.pone.0352332 (PMC13340848; doi:10.1371/journal.pone.0352332)
Supplement: Attachment — Submitted filename: Response to reviewers v2.docx [file pone.0352332.s010.docx]

Maastricht, The Netherlands

May 19, 2026

Dear dr. Giove,

Thank you for your e-mail on the 15^th^ of May regarding our revision of the manuscript entitled “Long-term multidimensional health status of individuals with and without post COVID-19 condition: a cross-sectional study” with manuscript ID: PONE-D-25-63012.

We appreciate the editor’s and reviewers’ consideration of our manuscript for publication in *PLOS ONE*. We have prepared a point-by-point response to all reviewer comments, which is provided in the accompanying rebuttal letter. In addition, we have revised the Data Availability Statement in the submission system to ensure compliance with journal requirements.

As no changes were made to the manuscript text itself, no revised manuscript file has been uploaded.

We hope that our responses are satisfactory and that the manuscript can now be considered for publication in *PLOS ONE*.

Kind regards,

The authors

**Comments editor**

The scientific content of the revised manuscript is sound, and I consider it ready for publication. However, the data availability statement is contradictory, and the manuscript does not provide clear information on how the data can be accessed.

The authors should carefully verify that their data sharing policy complies with PLOS ONE requirements. In general, data must be deposited in a public repository, and the corresponding link should be included in the manuscript. If public sharing is not possible (for example, due to legal restrictions), the reasons must be clearly stated, and a detailed procedure describing how the data can be accessed should be provided in the manuscript body.  If there are no legal restrictions preventing data sharing, stating that the data are “available from the corresponding author upon request” is not compliant with PLOS ONE data availability policies.

*We thank the editor for the positive evaluation of the scientific content of our revised manuscript and for highlighting the issue regarding the data availability statement. We agree that the previous wording may have been unclear and potentially contradictory. Therefore, we have revised the Data Availability Statement to ensure full compliance with the PLOS ONE data sharing policy. In addition, the dataset has now been uploaded as supporting information within the submission system to facilitate transparency, reproducibility, and open science practices.*

Journal Requirements:

1. If the reviewer comments include a recommendation to cite specific previously published works, please review and evaluate these publications to determine whether they are relevant and should be cited. There is no requirement to cite these works unless the editor has indicated otherwise.

*Not applicable. No reviewer recommendations regarding additional citations were provided.*

2. Please review your reference list to ensure that it is complete and correct. If you have cited papers that have been retracted, please include the rationale for doing so in the manuscript text, or remove these references and replace them with relevant current references. Any changes to the reference list should be mentioned in the rebuttal letter that accompanies your revised manuscript. If you need to cite a retracted article, indicate the article’s retracted status in the References list and also include a citation and full reference for the retraction notice.

*We have carefully reviewed the reference list and confirmed that it is complete and accurate. None of the cited references have been retracted; therefore, no further changes to the reference list were necessary.*

Reviewer's Responses to Questions

**Comments to the Author**

1. If the authors have adequately addressed your comments raised in a previous round of review and you feel that this manuscript is now acceptable for publication, you may indicate that here to bypass the “Comments to the Author” section, enter your conflict of interest statement in the “Confidential to Editor” section, and submit your "Accept" recommendation.

Reviewer #1: All comments have been addressed

Reviewer #2: All comments have been addressed

Reviewer #3: All comments have been addressed

2. Is the manuscript technically sound, and do the data support the conclusions?

The manuscript must describe a technically sound piece of scientific research with data that supports the conclusions. Experiments must have been conducted rigorously, with appropriate controls, replication, and sample sizes. The conclusions must be drawn appropriately based on the data presented.

Reviewer #1: Yes

Reviewer #2: Yes

Reviewer #3: Yes

3. Has the statistical analysis been performed appropriately and rigorously?

Reviewer #1: Yes

Reviewer #2: Yes

Reviewer #3: Yes

4. Have the authors made all data underlying the findings in their manuscript fully available?

The [PLOS Data policy](http://track.editorialmanager.com/CL0/http:%2F%2Fwww.plosone.org%2Fstatic%2Fpolicies.action%23sharing/1/010f019e292ad932-6c025012-fd29-47fa-8cf3-1a07760e7c93-000000/3XpmeWx8IdKC_utt-KXebFRq5vCWnAIdrKwdmBarUsc=258) requires authors to make all data underlying the findings described in their manuscript fully available without restriction, with rare exception (please refer to the Data Availability Statement in the manuscript PDF file). The data should be provided as part of the manuscript or its supporting information, or deposited to a public repository. For example, in addition to summary statistics, the data points behind means, medians and variance measures should be available. If there are restrictions on publicly sharing data—e.g. participant privacy or use of data from a third party—those must be specified.

Reviewer #1: Yes

Reviewer #2: Yes

Reviewer #3: No

5. Is the manuscript presented in an intelligible fashion and written in standard English?

PLOS ONE does not copyedit accepted manuscripts, so the language in submitted articles must be clear, correct, and unambiguous. Any typographical or grammatical errors should be corrected at revision, so please note any specific errors here.

Reviewer #1: Yes

Reviewer #2: Yes

Reviewer #3: Yes

6. Review Comments to the Author

Please use the space provided to explain your answers to the questions above. You may also include additional comments for the author, including concerns about dual publication, research ethics, or publication ethics. (Please upload your review as an attachment if it exceeds 20,000 characters)

Reviewer #1: The manuscript is readable and the methods are quite accurate. The results of the study are interesting and there are few studies on this subject, worldwide. I consider the article to be worth publishing.

*We sincerely thank the reviewer for the positive assessment of our manuscript, including the readability of the text, the appropriateness of the methods, and the relevance of the study topic. We appreciate the reviewer’s overall favorable evaluation and recommendation for publication.*

Reviewer #2: (No Response)

Reviewer #3: It is wonderful to receive the revised manuscript titled "Long-term multidimensional health status of individuals with and without post-COVID-19 condition: a cross-sectional study” (PONE-D-25-63012R1). The authors have appropriately addressed several important concerns, including PCC, potential confounding, causal interpretation, and clarity of writing. Thus, I recommend acceptance after minor revision. Overall, the manuscript is scientifically solid, clinically relevant, and methodologically transparent. However, a few relatively minor issues could be addressed before final acceptance. The data availability statement (“Datasets are available upon request”) may not fully comply with PLOS ONE's strict data-sharing policy and may require clarification or repository deposition. In addition, the study still relies on retrospective self-reported PCC classification, and the comparisons remain unadjusted for baseline differences such as hospitalization and comorbidity burden. Nevertheless, the authors now openly acknowledge these limitations, which substantially strengthen the manuscript.

*We sincerely thank the reviewer for the positive evaluation of our revised manuscript and for recognizing the efforts made to address the previously raised concerns regarding PCC classification, potential confounding, causal interpretation, and clarity of writing. We appreciate the reviewer’s acknowledgment that the transparent discussion of the study limitations has strengthened the manuscript.*

*Regarding the data availability statement, we have revised the statement to ensure compliance with the PLOS ONE data-sharing policy. In addition, the dataset has been uploaded as supporting information within the submission system to facilitate transparency and reproducibility.*

*The remaining limitations concerning retrospective self-reported PCC classification and unadjusted baseline differences are now explicitly acknowledged and discussed in the manuscript as important considerations when interpreting the findings.*

7. PLOS authors have the option to publish the peer review history of their article ([what does this mean?](https://track.editorialmanager.com/CL0/https:%2F%2Fjournals.plos.org%2Fplosone%2Fs%2Feditorial-and-peer-review-process%23loc-peer-review-history/1/010f019e292ad932-6c025012-fd29-47fa-8cf3-1a07760e7c93-000000/UkYC0XukacdGwuJMUGDSV1j4wvNazn7xEr-R9WPNyM8=258)). If published, this will include your full peer review and any attached files.


If you choose “no”, your identity will remain anonymous but your review may still be made public.

**Do you want your identity to be public for this peer review?** For information about this choice, including consent withdrawal, please see our [Privacy Policy](https://track.editorialmanager.com/CL0/https:%2F%2Fwww.plos.org%2Fprivacy-policy/1/010f019e292ad932-6c025012-fd29-47fa-8cf3-1a07760e7c93-000000/F9s4fZ5zYtFVWeyNFqsh2cV186yKTYjhPYj4ptYSWwc=258).

Reviewer #1: No

Reviewer #2: No

Reviewer #3: No
