# Supplementary material for: Long-term multidimensional health status of individuals with and without post COVID-19 condition: A cross-sectional study
Source: PLoS One. 2026 Jul 7;21(7):e0352332. doi: 10.1371/journal.pone.0352332 (PMC13340848; doi:10.1371/journal.pone.0352332)
Supplement: Peer review history

# Peer review: Decision Letter 0

**DOI:** 10.1371/journal.pone.0352332.r001

**Authors:** Jiajie She

13 Feb 2026

PONE-D-25-63012

Long-term multidimensional health status of individuals with and without post COVID-19 condition: a cross-sectional study

PLOS One

Dear Dr. Gach,

Thank you for submitting your manuscript to PLOS ONE. After careful consideration, we feel that it has merit but does not fully meet PLOS ONE’s publication criteria as it currently stands. Therefore, we invite you to submit a revised version of the manuscript that addresses the points raised during the review process.

Please submit your revised manuscript by Mar 30 2026 11:59PM. If you will need more time than this to complete your revisions, please reply to this message or contact the journal office at plosone@plos.org. When you're ready to submit your revision, log on to https://www.editorialmanager.com/pone/ and select the 'Submissions Needing Revision' folder to locate your manuscript file.

Please include the following items when submitting your revised manuscript:

- A letter that responds to each point raised by the academic editor and reviewer(s). You should upload this letter as a separate file labeled 'Response to Reviewers'.
- A marked-up copy of your manuscript that highlights changes made to the original version. You should upload this as a separate file labeled 'Revised Manuscript with Track Changes'.
- An unmarked version of your revised paper without tracked changes. You should upload this as a separate file labeled 'Manuscript'.

-->If you would like to make changes to your financial disclosure, please include your updated statement in your cover letter. Guidelines for resubmitting your figure files are available below the reviewer comments at the end of this letter.

If applicable, we recommend that you deposit your laboratory protocols in protocols.io to enhance the reproducibility of your results. Protocols.io assigns your protocol its own identifier (DOI) so that it can be cited independently in the future. For instructions see: https://journals.plos.org/plosone/s/submission-guidelines#loc-laboratory-protocols. Additionally, PLOS ONE offers an option for publishing peer-reviewed Lab Protocol articles, which describe protocols hosted on protocols.io. Read more information on sharing protocols at https://plos.org/protocols?utm_medium=editorial-email&utm_source=authorletters&utm_campaign=protocols.

We look forward to receiving your revised manuscript.

Kind regards,

Jiajie She

Academic Editor

PLOS One

Journal Requirements:

When submitting your revision, we need you to address these additional requirements.

1. Please ensure that your manuscript meets PLOS ONE's style requirements, including those for file naming. The PLOS ONE style templates can be found at

https://journals.plos.org/plosone/s/file?id=wjVg/PLOSOne_formatting_sample_main_body.pdf and https://journals.plos.org/plosone/s/file?id=ba62/PLOSOne_formatting_sample_title_authors_affiliations.pdf

2. Thank you for stating in your Funding Statement:

[Funding statement: This study was supported by a Research Fellowship from the European Society of Clinical Nutrition and Metabolism (ESPEN) awarded to Rosanne J.H.C.G. Beijers, and by a ZonMw grant (project number 100430040211004) awarded to Hester A. Gietema, and Annemie M.W.J. Schols funded this work. The funders had no role in study design, data collection and analysis, decision to publish, or preparation of the manuscript.].

Please provide an amended statement that declares *all* the funding or sources of support (whether external or internal to your organization) received during this study, as detailed online in our guide for authors at http://journals.plos.org/plosone/s/submit-now.  Please also include the statement “There was no additional external funding received for this study.” in your updated Funding Statement.

Please include your amended Funding Statement within your cover letter. We will change the online submission form on your behalf.

3. Thank you for stating the following in your manuscript:

[This study was supported by a Research Fellowship from the European Society of Clinical Nutrition and Metabolism (ESPEN) awarded to Rosanne J.H.C.G. Beijers, and by a ZonMw grant (project number 100430040211004) awarded to Hester A. Gietema, and Annemie M.W.J. Schols funded this work. The funders had no role in study design, data collection and analysis, decision to publish, or preparation of the manuscript.]

We note that you have provided funding information that is currently declared in your Funding Statement. However, funding information should not appear in the Acknowledgments section or other areas of your manuscript. We will only publish funding information present in the Funding Statement section of the online submission form.

Please remove any funding-related text from the manuscript and let us know how you would like to update your Funding Statement. Currently, your Funding Statement reads as follows:

[Funding statement: This study was supported by a Research Fellowship from the European Society of Clinical Nutrition and Metabolism (ESPEN) awarded to Rosanne J.H.C.G. Beijers, and by a ZonMw grant (project number 100430040211004) awarded to Hester A. Gietema, and Annemie M.W.J. Schols funded this work. The funders had no role in study design, data collection and analysis, decision to publish, or preparation of the manuscript.]

Please include your amended statements within your cover letter; we will change the online submission form on your behalf.

4. Thank you for stating the following in the Competing Interests section:

[I have read the journal's policy and the authors of this manuscript have the following competing interests: J. van den Bergh has research funding from POROUS, and consultancy from UCB and AMGEN, which was not related to this work. No competing interests exist for the other authors.].

We note that you received funding from commercial sources: POROUS, UCB, and AMGEN.

Please provide an amended Competing Interests Statement that explicitly states this commercial funder, along with any other relevant declarations relating to employment, consultancy, patents, products in development, marketed products, etc.

Within this Competing Interests Statement, please confirm that this does not alter your adherence to all PLOS ONE policies on sharing data and materials by including the following statement: "This does not alter our adherence to PLOS ONE policies on sharing data and materials.” (as detailed online in our guide for authors http://journals.plos.org/plosone/s/competing-interests).  If there are restrictions on sharing of data and/or materials, please state these. Please note that we cannot proceed with consideration of your article until this information has been declared.

Please include your amended Competing Interests Statement within your cover letter. We will change the online submission form on your behalf.

5. One of the noted authors is a group or consortium [COVID CLIMATE Consortium]. In addition to naming the author group, please list the individual authors and affiliations within this group in the acknowledgments section of your manuscript. Please also indicate clearly a lead author for this group along with a contact email address.

6. We note that Figures 2 and 6 in your submission contain images which may be copyrighted. All PLOS content is published under the Creative Commons Attribution License (CC BY 4.0), which means that the manuscript, images, and Supporting Information files will be freely available online, and any third party is permitted to access, download, copy, distribute, and use these materials in any way, even commercially, with proper attribution. For more information, see our copyright guidelines: http://journals.plos.org/plosone/s/licenses-and-copyright.

We require you to either (1) present written permission from the copyright holder to publish these figures specifically under the CC BY 4.0 license, or (2) remove the figures from your submission:

1. You may seek permission from the original copyright holder of Figures 2 and 6 to publish the content specifically under the CC BY 4.0 license.

We recommend that you contact the original copyright holder with the Content Permission Form (http://journals.plos.org/plosone/s/file?id=7c09/content-permission-form.pdf) and the following text:

“I request permission for the open-access journal PLOS ONE to publish XXX under the Creative Commons Attribution License (CCAL) CC BY 4.0 (http://creativecommons.org/licenses/by/4.0/). Please be aware that this license allows unrestricted use and distribution, even commercially, by third parties. Please reply and provide explicit written permission to publish XXX under a CC BY license and complete the attached form.”

Please upload the completed Content Permission Form or other proof of granted permissions as an "Other" file with your submission.

In the figure caption of the copyrighted figure, please include the following text: “Reprinted from [ref] under a CC BY license, with permission from [name of publisher], original copyright [original copyright year].”

2. If you are unable to obtain permission from the original copyright holder to publish these figures under the CC BY 4.0 license or if the copyright holder’s requirements are incompatible with the CC BY 4.0 license, please either i) remove the figure or ii) supply a replacement figure that complies with the CC BY 4.0 license. Please check copyright information on all replacement figures and update the figure caption with source information. If applicable, please specify in the figure caption text when a figure is similar but not identical to the original image and is therefore for illustrative purposes only.

7. Please include captions for your Supporting Information files at the end of your manuscript, and update any in-text citations to match accordingly. Please see our Supporting Information guidelines for more information: http://journals.plos.org/plosone/s/supporting-information.

If the reviewer comments include a recommendation to cite specific previously published works, please review and evaluate these publications to determine whether they are relevant and should be cited. There is no requirement to cite these works unless the editor has indicated otherwise.

Please review your reference list to ensure that it is complete and correct. If you have cited papers that have been retracted, please include the rationale for doing so in the manuscript text, or remove these references and replace them with relevant current references. Any changes to the reference list should be mentioned in the rebuttal letter that accompanies your revised manuscript. If you need to cite a retracted article, indicate the article’s retracted status in the References list and also include a citation and full reference for the retraction notice.

[Note: HTML markup is below. Please do not edit.]

Reviewers' comments:

Reviewer's Responses to Questions

**Comments to the Author**

1. Is the manuscript technically sound, and do the data support the conclusions?

The manuscript must describe a technically sound piece of scientific research with data that supports the conclusions. Experiments must have been conducted rigorously, with appropriate controls, replication, and sample sizes. The conclusions must be drawn appropriately based on the data presented.

Reviewer #1: Yes

Reviewer #2: Yes

Reviewer #3: Yes

Reviewer #4: Yes

**********

2. Has the statistical analysis been performed appropriately and rigorously?

Reviewer #1: Yes

Reviewer #2: Yes

Reviewer #3: Yes

Reviewer #4: Yes

**********

3. Have the authors made all data underlying the findings in their manuscript fully available?

The PLOS Data policy requires authors to make all data underlying the findings described in their manuscript fully available without restriction, with rare exception (please refer to the Data Availability Statement in the manuscript PDF file). The data should be provided as part of the manuscript or its supporting information, or deposited to a public repository. For example, in addition to summary statistics, the data points behind means, medians and variance measures should be available. If there are restrictions on publicly sharing data—e.g. participant privacy or use of data from a third party—those must be specified.

Reviewer #1: Yes

Reviewer #2: Yes

Reviewer #3: Yes

Reviewer #4: Yes

**********

4. Is the manuscript presented in an intelligible fashion and written in standard English?

PLOS ONE does not copyedit accepted manuscripts, so the language in submitted articles must be clear, correct, and unambiguous. Any typographical or grammatical errors should be corrected at revision, so please note any specific errors here.

Reviewer #1: Yes

Reviewer #2: Yes

Reviewer #3: Yes

Reviewer #4: Yes

**********

5. Review Comments to the Author

Please use the space provided to explain your answers to the questions above. You may also include additional comments for the author, including concerns about dual publication, research ethics, or publication ethics. (Please upload your review as an attachment if it exceeds 20,000 characters)

Reviewer #1: This study presents a technically sound, well-written analysis that demonstrates differences in COVID-19 severity. It is an exciting article to publish. The title is Long-term multidimensional health status of individuals with and without post COVID-19 condition: a cross-sectional study, and the ID number is (PONE-D-25-63012). The authors should run the manuscript through Grammarly to ensure spelling and grammar are correct before publishing the article.

Reviewer #2: Major comments

1. The follow-up interval in this study introduces a potential risk of recall bias among participants. The rationale for selecting a minimum follow-up duration of one year should be more clearly justified, and the reasons for not applying the WHO symptom-duration criteria for PCC (minimum three months) at the time of assessment should be explicitly explained.

2. In addition, the delayed, self-reported classification of PCC may introduce result-driven classification bias due to increased recall bias. This temporal gap between the multidimensional health assessment and PCC classification may affect the internal validity of group comparisons and correlation analyses. The authors are encouraged to clarify whether symptom status reported at follow-up accurately reflected participants’ health status at the time of objective testing.

3. Lack of adjustment for baseline group differences (e.g., CCI, hospitalization) should be noted as a limitation

Minor Comments

1. Add a short note on clinical and rehabilitation implications.

2. Correct minor formatting and ensure consistent terminology and supplementary figure referencing.

Reviewer #3: Reviewer

It is an honor to review the manuscript entitled "Long-term multidimensional health status of individuals with and without post-COVID-19 condition: a cross-sectional study” (PONE-D-25-63012). I have carefully reviewed this manuscript and found that it addresses high-quality, clinically relevant issues, employs strong methodology, and conveys an important message. It presents a well-designed, comprehensive multidimensional assessment of long-term health outcomes in individuals with and without self-reported post-COVID-19 condition (PCC) at ≥1 year after SARS-CoV-2 infection. The topic is timely, clinically relevant, and aligned with PLOS ONE’s scope. The study’s strengths include the breadth of objective and subjective assessments, inclusion of both hospitalized and non-hospitalized patients, and the direct comparison between PCC and non-PCC groups. However, minor issues should be addressed to strengthen the manuscript, particularly regarding the PCC definition, potential confounding, causal interpretation, and writing style, in accordance with PLOS ONE’s guidelines. After this revision, I support accepting this manuscript in PLOS ONE.

Manor’s suggestion and revision

1. As I understand it, PLOS ONE’s guidelines do not require subtitles in the abstract. Instead, the abstract should be a concise summary of fewer than 300 words that covers the study's objective, methods (briefly), key results, and significance, without citations or excessive abbreviations.

2. I have a major concern about this. Please read this carefully. It may help strengthen your manuscripts. Post-COVID-19 condition (PCC) was defined solely on the basis of retrospective self-report during a follow-up phone call, using three yes/no questions. This approach may introduce recall bias, subjectivity, and potential circularity between symptom reporting and outcome measures. This limitation should be explicitly acknowledged in both the Methods and Discussion sections. The authors should also clarify why a standardized PCC definition, such as WHO-based symptom criteria or duration thresholds, was not applied. Where possible, sensitivity analyses using symptom burden thresholds (e.g., fatigue, dyspnea, or cognitive complaints) could strengthen the findings, and causal language should be carefully revised to reflect associations rather than causation.

3. The author has shown that there were significant baseline differences between the PCC and non-PCC groups, including the Charlson Comorbidity Index, smoking status, hospitalization rates, and COVID-19 wave distribution, which may confound comparisons of health-related quality of life and physical performance. To address this, adjusted analyses, such as multivariable regression controlling for age, sex, comorbidity burden, hospitalization status, and time since infection, are recommended. At a minimum, please clearly state that the results are based on unadjusted comparisons and interpret the findings with appropriate caution.

4. In the abstract, at the end of Lines 56-57, the author states that this suggests current objective testing methods could not adequately reflect patients’ perceived health burden in PCC, a claim that is not fully supported by the cross-sectional study design. The findings indicate a discordance between subjective health burden and currently available objective assessments in individuals with post-COVID-19 condition.

Reviewer #4: Authors present a cross-sectional assessment of long-term, multidimensional health status in individuals with and without self-reported post COVID-19 condition (PCC), evaluated at least one year after SARS-CoV-2 infection. The authors combine subjective and objective measures across multiple domains, including health-related quality of life, symptoms, psychological and social wellbeing, pulmonary and metabolic health, muscle strength, physical capability, and cognition. The study addresses a relevant clinical question and provides a broad overview of how perceived health burden relates to objectively measured outcomes in this population. While the overall design and structure are clear, some aspects—particularly the operational definition of PCC, the characteristics of the comparison group, and the interpretation of discrepancies between subjective and objective findings—would benefit from additional clarification. With these points addressed, I believe the manuscript would be appropriate for publication.

Points that would benefit from clarification:

1. PCC is defined here based on self-report, including the follow-up call. I would like some clarification on how misclassification was handled. Were alternative diagnoses actively excluded? How closely does your operational definition align with the WHO criteria in terms of symptom onset and duration (≥3 months after infection, lasting ≥2 months)? A clearer mapping of your definition to current PCC guidance would help.

2. Participants were recruited from hospitalized cohorts and from a post-COVID outpatient clinic, which may already select for individuals with ongoing complaints. I am not fully clear on how the “without PCC” group should be interpreted in this context. What were the main reasons these participants attended the assessment, and how confident can we be that this group represents a true post-infection comparator rather than a clinically selected population?

3. An interesting finding is that individuals with PCC had fewer comorbidities and were less often hospitalized than those without PCC, which contrasts with part of the existing literature. This raises the question of potential confounding or selection effects. Were key outcomes (e.g., HRQoL, fatigue, sleep quality, MIP, 6MWD%pred) examined with adjustment for relevant variables such as age, sex, time since infection, hospitalization/ICU, CCI, smoking status, and infection wave or vaccination period? Even a brief adjusted or sensitivity analysis would make the interpretation more robust.

4. You report clear differences in subjective cognitive complaints (CFQ) but not in objective screening (MoCA). I wonder whether MoCA may simply lack sensitivity for the type of cognitive difficulties reported by PCC patients (e.g., attention, executive function, fatigue-related issues). Some discussion on measurement sensitivity, and on whether fatigue, sleep disturbance, or mood could be mediating subjective cognitive complaints, would help frame this discrepancy.

If you address these points, the overall interpretation will be clearer.

**********

6. PLOS authors have the option to publish the peer review history of their article (what does this mean?). If published, this will include your full peer review and any attached files.

If you choose “no”, your identity will remain anonymous but your review may still be made public.

**Do you want your identity to be public for this peer review?**  For information about this choice, including consent withdrawal, please see our Privacy Policy.

Reviewer #1: No

Reviewer #2: No

Reviewer #3: No

Reviewer #4: No

**********

[NOTE: If reviewer comments were submitted as an attachment file, they will be attached to this email and accessible via the submission site. Please log into your account, locate the manuscript record, and check for the action link "View Attachments". If this link does not appear, there are no attachment files.]

To ensure your figures meet our technical requirements, please review our figure guidelines: https://journals.plos.org/plosone/s/figures

You may also use PLOS’s free figure tool, NAAS, to help you prepare publication quality figures: https://journals.plos.org/plosone/s/figures#loc-tools-for-figure-preparation.

NAAS will assess whether your figures meet our technical requirements by comparing each figure against our figure specifications.

Attachment Submitted filename: Reviewer comments.pdf

# Author comment: Author response to Decision Letter 1

**DOI:** 10.1371/journal.pone.0352332.r002

30 Mar 2026

We have attached the response to the reviewer and editor comments as an attached file under "response to reviewers"

Attachment Submitted filename: Response to reviewers.docx

# Peer review: Decision Letter 1

**DOI:** 10.1371/journal.pone.0352332.r003

**Authors:** Jiajie She, Federico Giove

14 May 2026

PONE-D-25-63012R1

Long-term multidimensional health status of individuals with and without post COVID-19 condition: a cross-sectional study

PLOS One

Dear Dr. Gach,

Thank you for submitting your manuscript to PLOS ONE. After careful consideration, we feel that it has merit but does not fully meet PLOS ONE’s publication criteria as it currently stands. Therefore, we invite you to submit a revised version of the manuscript that addresses the points raised during the review process.

====================================

The scientific content of the revised manuscript is sound, and I consider it ready for publication. However, the data availability statement is contradictory, and the manuscript does not provide clear information on how the data can be accessed.

The authors should carefully verify that their data sharing policy complies with PLOS ONE requirements. In general, data must be deposited in a public repository, and the corresponding link should be included in the manuscript. If public sharing is not possible (for example, due to legal restrictions), the reasons must be clearly stated, and a detailed procedure describing how the data can be accessed should be provided in the manuscript body.  If there are no legal restrictions preventing data sharing, stating that the data are “available from the corresponding author upon request” is not compliant with PLOS ONE data availability policies.

====================================

Please submit your revised manuscript by Jun 28 2026 11:59PM. If you will need more time than this to complete your revisions, please reply to this message or contact the journal office at plosone@plos.org. When you're ready to submit your revision, log on to https://www.editorialmanager.com/pone/ and select the 'Submissions Needing Revision' folder to locate your manuscript file.

Please include the following items when submitting your revised manuscript:

- A letter that responds to each point raised by the academic editor and reviewer(s). You should upload this letter as a separate file labeled 'Response to Reviewers'.
- A marked-up copy of your manuscript that highlights changes made to the original version. You should upload this as a separate file labeled 'Revised Manuscript with Track Changes'.
- An unmarked version of your revised paper without tracked changes. You should upload this as a separate file labeled 'Manuscript'.

If you would like to make changes to your financial disclosure, please include your updated statement in your cover letter. Guidelines for resubmitting your figure files are available below the reviewer comments at the end of this letter.

If applicable, we recommend that you deposit your laboratory protocols in protocols.io to enhance the reproducibility of your results. Protocols.io assigns your protocol its own identifier (DOI) so that it can be cited independently in the future. For instructions see: https://journals.plos.org/plosone/s/submission-guidelines#loc-laboratory-protocols. Additionally, PLOS ONE offers an option for publishing peer-reviewed Lab Protocol articles, which describe protocols hosted on protocols.io. Read more information on sharing protocols at https://plos.org/protocols?utm_medium=editorial-email&utm_source=authorletters&utm_campaign=protocols.

As the corresponding author, your ORCID iD is verified in the submission system and will appear in the published article. PLOS supports the use of ORCID, and we encourage all coauthors to register for an ORCID iD and use it as well. Please encourage your coauthors to verify their ORCID iD within the submission system before final acceptance, as unverified ORCID iDs will not appear in the published article. *Only*  the individual author can complete the verification step; PLOS staff *cannot*  verify ORCID iDs on behalf of authors.

We look forward to receiving your revised manuscript.

Kind regards,

Federico Giove, PhD

Academic Editor

PLOS One

Journal Requirements:

1. If the reviewer comments include a recommendation to cite specific previously published works, please review and evaluate these publications to determine whether they are relevant and should be cited. There is no requirement to cite these works unless the editor has indicated otherwise.

2. Please review your reference list to ensure that it is complete and correct. If you have cited papers that have been retracted, please include the rationale for doing so in the manuscript text, or remove these references and replace them with relevant current references. Any changes to the reference list should be mentioned in the rebuttal letter that accompanies your revised manuscript. If you need to cite a retracted article, indicate the article’s retracted status in the References list and also include a citation and full reference for the retraction notice.

[Note: HTML markup is below. Please do not edit.]

Reviewers' comments:

Reviewer's Responses to Questions

**Comments to the Author**

1. If the authors have adequately addressed your comments raised in a previous round of review and you feel that this manuscript is now acceptable for publication, you may indicate that here to bypass the “Comments to the Author” section, enter your conflict of interest statement in the “Confidential to Editor” section, and submit your "Accept" recommendation.

Reviewer #1: All comments have been addressed

Reviewer #2: All comments have been addressed

Reviewer #3: All comments have been addressed

**********

2. Is the manuscript technically sound, and do the data support the conclusions?

The manuscript must describe a technically sound piece of scientific research with data that supports the conclusions. Experiments must have been conducted rigorously, with appropriate controls, replication, and sample sizes. The conclusions must be drawn appropriately based on the data presented.

Reviewer #1: Yes

Reviewer #2: Yes

Reviewer #3: Yes

**********

3. Has the statistical analysis been performed appropriately and rigorously?

Reviewer #1: Yes

Reviewer #2: Yes

Reviewer #3: Yes

**********

4. Have the authors made all data underlying the findings in their manuscript fully available?

The PLOS Data policy requires authors to make all data underlying the findings described in their manuscript fully available without restriction, with rare exception (please refer to the Data Availability Statement in the manuscript PDF file). The data should be provided as part of the manuscript or its supporting information, or deposited to a public repository. For example, in addition to summary statistics, the data points behind means, medians and variance measures should be available. If there are restrictions on publicly sharing data—e.g. participant privacy or use of data from a third party—those must be specified.

Reviewer #1: Yes

Reviewer #2: Yes

Reviewer #3: No

**********

5. Is the manuscript presented in an intelligible fashion and written in standard English?

PLOS ONE does not copyedit accepted manuscripts, so the language in submitted articles must be clear, correct, and unambiguous. Any typographical or grammatical errors should be corrected at revision, so please note any specific errors here.

Reviewer #1: Yes

Reviewer #2: Yes

Reviewer #3: Yes

**********

6. Review Comments to the Author

Please use the space provided to explain your answers to the questions above. You may also include additional comments for the author, including concerns about dual publication, research ethics, or publication ethics. (Please upload your review as an attachment if it exceeds 20,000 characters)

Reviewer #1: The manuscript is readable and the methods are quite accurate. The results of the study are interesting and there are few studies on this subject, worldwide. I consider the article to be worth publishing.

Reviewer #2: (No Response)

Reviewer #3: It is wonderful to receive the revised manuscript titled "Long-term multidimensional health status of individuals with and without post-COVID-19 condition: a cross-sectional study” (PONE-D-25-63012R1). The authors have appropriately addressed several important concerns, including PCC, potential confounding, causal interpretation, and clarity of writing. Thus, I recommend acceptance after minor revision. Overall, the manuscript is scientifically solid, clinically relevant, and methodologically transparent. However, a few relatively minor issues could be addressed before final acceptance. The data availability statement (“Datasets are available upon request”) may not fully comply with PLOS ONE's strict data-sharing policy and may require clarification or repository deposition. In addition, the study still relies on retrospective self-reported PCC classification, and the comparisons remain unadjusted for baseline differences such as hospitalization and comorbidity burden. Nevertheless, the authors now openly acknowledge these limitations, which substantially strengthen the manuscript.

**********

7. PLOS authors have the option to publish the peer review history of their article (what does this mean?). If published, this will include your full peer review and any attached files.

If you choose “no”, your identity will remain anonymous but your review may still be made public.

**Do you want your identity to be public for this peer review?**  For information about this choice, including consent withdrawal, please see our Privacy Policy.

Reviewer #1: No

Reviewer #2: No

Reviewer #3: No

**********

[NOTE: If reviewer comments were submitted as an attachment file, they will be attached to this email and accessible via the submission site. Please log into your account, locate the manuscript record, and check for the action link "View Attachments". If this link does not appear, there are no attachment files.]

To ensure your figures meet our technical requirements, please review our figure guidelines: https://journals.plos.org/plosone/s/figures

You may also use PLOS’s free figure tool, NAAS, to help you prepare publication quality figures: https://journals.plos.org/plosone/s/figures#loc-tools-for-figure-preparation.

NAAS will assess whether your figures meet our technical requirements by comparing each figure against our figure specifications.

Attachment Submitted filename: Reviewer 3 comments.pdf

# Author comment: Author response to Decision Letter 2

**DOI:** 10.1371/journal.pone.0352332.r004

26 May 2026

Maastricht, The Netherlands

May 19, 2026

Dear dr. Giove,

Thank you for your e-mail on the 15th of May regarding our revision of the manuscript entitled “Long-term multidimensional health status of individuals with and without post COVID-19 condition: a cross-sectional study” with manuscript ID: PONE-D-25-63012.

We appreciate the editor’s and reviewers’ consideration of our manuscript for publication in PLOS ONE. We have prepared a point-by-point response to all reviewer comments, which is provided in the accompanying rebuttal letter. In addition, we have revised the Data Availability Statement in the submission system to ensure compliance with journal requirements.

As no changes were made to the manuscript text itself, no revised manuscript file has been uploaded.

We hope that our responses are satisfactory and that the manuscript can now be considered for publication in PLOS ONE.

Kind regards,

The authors

Comments editor

The scientific content of the revised manuscript is sound, and I consider it ready for publication. However, the data availability statement is contradictory, and the manuscript does not provide clear information on how the data can be accessed.

The authors should carefully verify that their data sharing policy complies with PLOS ONE requirements. In general, data must be deposited in a public repository, and the corresponding link should be included in the manuscript. If public sharing is not possible (for example, due to legal restrictions), the reasons must be clearly stated, and a detailed procedure describing how the data can be accessed should be provided in the manuscript body. If there are no legal restrictions preventing data sharing, stating that the data are “available from the corresponding author upon request” is not compliant with PLOS ONE data availability policies.

We thank the editor for the positive evaluation of the scientific content of our revised manuscript and for highlighting the issue regarding the data availability statement. We agree that the previous wording may have been unclear and potentially contradictory. Therefore, we have revised the Data Availability Statement to ensure full compliance with the PLOS ONE data sharing policy. In addition, the dataset has now been uploaded as supporting information within the submission system to facilitate transparency, reproducibility, and open science practices.

Journal Requirements:

1. If the reviewer comments include a recommendation to cite specific previously published works, please review and evaluate these publications to determine whether they are relevant and should be cited. There is no requirement to cite these works unless the editor has indicated otherwise.

Not applicable. No reviewer recommendations regarding additional citations were provided.

2. Please review your reference list to ensure that it is complete and correct. If you have cited papers that have been retracted, please include the rationale for doing so in the manuscript text, or remove these references and replace them with relevant current references. Any changes to the reference list should be mentioned in the rebuttal letter that accompanies your revised manuscript. If you need to cite a retracted article, indicate the article’s retracted status in the References list and also include a citation and full reference for the retraction notice.

We have carefully reviewed the reference list and confirmed that it is complete and accurate. None of the cited references have been retracted; therefore, no further changes to the reference list were necessary.

Reviewer's Responses to Questions

Comments to the Author

1. If the authors have adequately addressed your comments raised in a previous round of review and you feel that this manuscript is now acceptable for publication, you may indicate that here to bypass the “Comments to the Author” section, enter your conflict of interest statement in the “Confidential to Editor” section, and submit your "Accept" recommendation.

Reviewer #1: All comments have been addressed

Reviewer #2: All comments have been addressed

Reviewer #3: All comments have been addressed

________________________________________

2. Is the manuscript technically sound, and do the data support the conclusions?

The manuscript must describe a technically sound piece of scientific research with data that supports the conclusions. Experiments must have been conducted rigorously, with appropriate controls, replication, and sample sizes. The conclusions must be drawn appropriately based on the data presented.

Reviewer #1: Yes

Reviewer #2: Yes

Reviewer #3: Yes

________________________________________

3. Has the statistical analysis been performed appropriately and rigorously?

Reviewer #1: Yes

Reviewer #2: Yes

Reviewer #3: Yes

________________________________________

4. Have the authors made all data underlying the findings in their manuscript fully available?

The PLOS Data policy requires authors to make all data underlying the findings described in their manuscript fully available without restriction, with rare exception (please refer to the Data Availability Statement in the manuscript PDF file). The data should be provided as part of the manuscript or its supporting information, or deposited to a public repository. For example, in addition to summary statistics, the data points behind means, medians and variance measures should be available. If there are restrictions on publicly sharing data—e.g. participant privacy or use of data from a third party—those must be specified.

Reviewer #1: Yes

Reviewer #2: Yes

Reviewer #3: No

________________________________________

5. Is the manuscript presented in an intelligible fashion and written in standard English?

PLOS ONE does not copyedit accepted manuscripts, so the language in submitted articles must be clear, correct, and unambiguous. Any typographical or grammatical errors should be corrected at revision, so please note any specific errors here.

Reviewer #1: Yes

Reviewer #2: Yes

Reviewer #3: Yes

________________________________________

6. Review Comments to the Author

Please use the space provided to explain your answers to the questions above. You may also include additional comments for the author, including concerns about dual publication, research ethics, or publication ethics. (Please upload your review as an attachment if it exceeds 20,000 characters)

Reviewer #1: The manuscript is readable and the methods are quite accurate. The results of the study are interesting and there are few studies on this subject, worldwide. I consider the article to be worth publishing.

We sincerely thank the reviewer for the positive assessment of our manuscript, including the readability of the text, the appropriateness of the methods, and the relevance of the study topic. We appreciate the reviewer’s overall favorable evaluation and recommendation for publication.

Reviewer #2: (No Response)

Reviewer #3: It is wonderful to receive the revised manuscript titled "Long-term multidimensional health status of individuals with and without post-COVID-19 condition: a cross-sectional study” (PONE-D-25-63012R1). The authors have appropriately addressed several important concerns, including PCC, potential confounding, causal interpretation, and clarity of writing. Thus, I recommend acceptance after minor revision. Overall, the manuscript is scientifically solid, clinically relevant, and methodologically transparent. However, a few relatively minor issues could be addressed before final acceptance. The data availability statement (“Datasets are available upon request”) may not fully comply with PLOS ONE's strict data-sharing policy and may require clarification or repository deposition. In addition, the study still relies on retrospective self-reported PCC classification, and the comparisons remain unadjusted for baseline differences such as hospitalization and comorbidity burden. Nevertheless, the authors now openly acknowledge these limitations, which substantially strengthen the manuscript.

We sincerely thank the reviewer for the positive evaluation of our revised manuscript and for recognizing the efforts made to address the previously raised concerns regarding PCC classification, potential confounding, causal interpretation, and clarity of writing. We appreciate the reviewer’s acknowledgment that the transparent discussion of the study limitations has strengthened the manuscript.

Regarding the data availability statement, we have revised the statement to ensure compliance with the PLOS ONE data-sharing policy. In addition, the dataset has been uploaded as supporting information within the submission system to facilitate transparency and reproducibility.

The remaining limitations concerning retrospective self-reported PCC classification and unadjusted baseline differences are now explicitly acknowledged and discussed in the manuscript as important considerations when interpreting the findings.

________________________________________

7. PLOS authors have the option to publish the peer review history of their article (what does this mean?). If published, this will include your full peer review and any attached files.

If you choose “no”, your identity will remain anonymous but your review may still be made public.

Do you want your identity to be public for this peer review? For information about this choice, including consent withdrawal, please see our Privacy Policy.

Reviewer #1: No

Reviewer #2: No

Reviewer #3: No

Attachment Submitted filename: Response to reviewers v2.docx

# Editor report: Decision Letter 2

**DOI:** 10.1371/journal.pone.0352332.r005

**Authors:** Jiajie She, Federico Giove, Federico Giove

10 Jun 2026

Long-term multidimensional health status of individuals with and without post COVID-19 condition: a cross-sectional study

PONE-D-25-63012R2

Dear Dr. Gach,

We’re pleased to inform you that your manuscript has been judged scientifically suitable for publication and will be formally accepted for publication once it meets all outstanding technical requirements.

Within one week, you’ll receive an e-mail detailing the required amendments. When these have been addressed, you’ll receive a formal acceptance letter and your manuscript will be scheduled for publication.

An invoice will be generated when your article is formally accepted. Please note, if your institution has a publishing partnership with PLOS and your article meets the relevant criteria, all or part of your publication costs will be covered. Please make sure your user information is up-to-date by logging into Editorial Manager at Editorial Manager® and clicking the ‘Update My Information' link at the top of the page. For questions related to billing, please contact billing support.

If your institution or institutions have a press office, please notify them about your upcoming paper to help maximize its impact. If they’ll be preparing press materials, please inform our press team as soon as possible -- no later than 48 hours after receiving the formal acceptance. Your manuscript will remain under strict press embargo until 2 pm Eastern Time on the date of publication. For more information, please contact onepress@plos.org.

Kind regards,

Federico Giove, PhD

Academic Editor

PLOS One

Additional Editor Comments (optional):

Reviewers' comments:

# Editor report: Acceptance letter

**DOI:** 10.1371/journal.pone.0352332.r006

**Authors:** Jiajie She, Federico Giove, Federico Giove

PONE-D-25-63012R2

PLOS One

Dear Dr. Gach,

I'm pleased to inform you that your manuscript has been deemed suitable for publication in PLOS One. Congratulations! Your manuscript is now being handed over to our production team.

At this stage, our production department will prepare your paper for publication. This includes ensuring the following:

* All references, tables, and figures are properly cited

* All relevant supporting information is included in the manuscript submission,

* There are no issues that prevent the paper from being properly typeset

You will receive further instructions from the production team, including instructions on how to review your proof when it is ready. Please keep in mind that we are working through a large volume of accepted articles, so please give us a few days to review your paper and let you know the next and final steps.

Lastly, if your institution or institutions have a press office, please let them know about your upcoming paper now to help maximize its impact. If they'll be preparing press materials, please inform our press team within the next 48 hours. Your manuscript will remain under strict press embargo until 2 pm Eastern Time on the date of publication. For more information, please contact onepress@plos.org.

You will receive an invoice from PLOS for your publication fee after your manuscript has reached the completed accept phase. If you receive an email requesting payment before acceptance or for any other service, this may be a phishing scheme. Learn how to identify phishing emails and protect your accounts at https://explore.plos.org/phishing.

If we can help with anything else, please email us at customercare@plos.org.

Thank you for submitting your work to PLOS ONE and supporting open access.

Kind regards,

PLOS ONE Editorial Office Staff

on behalf of

Dr. Federico Giove

Academic Editor

PLOS One
